# Current Progress in CNS Imaging of Myotonic Dystrophy

**DOI:** 10.3389/fneur.2018.00646

**Published:** 2018-08-21

**Authors:** Martina Minnerop, Carla Gliem, Cornelia Kornblum

**Affiliations:** ^1^Institute of Neuroscience and Medicine (INM-1), Research Center Juelich, Juelich, Germany; ^2^Department of Neurology and Institute of Clinical Neuroscience and Medical Psychology, Medical Faculty, Center for Movement Disorders and Neuromodulation, Heinrich-Heine University, Düsseldorf, Germany; ^3^Department of Neurology, University Hospital of Bonn, Bonn, Germany; ^4^Center for Rare Diseases Bonn (ZSEB), University Hospital of Bonn, Bonn, Germany

**Keywords:** myotonic dystrophy, neuroimaging, MRI, PET, VBM, DTI, fMRI, brain

## Abstract

Neuroimaging in myotonic dystrophies provided a major contribution to the insight into brain involvement which is highly prevalent in these multisystemic disorders. Particular in Myotonic Dystrophy Type 1, conventional MRI first revealed hyperintense white matter lesions, predominantly localized in the anterior temporal lobe. Brain atrophy and ventricle enlargement were additional early findings already described almost 30 years ago. Since then, more advanced and sophisticated imaging methods have been applied in Myotonic Dystrophy Types 1 and 2. Involvement of actually normal appearing white matter and widespread cortical affection in PET studies were key results toward the recognition of diffuse and not only focally localized brain pathology *in vivo*. Later, structural abnormalities of both, gray and white matter, have been found in both forms of the disorder, albeit more prominent in myotonic dystrophy type 1. In Type 1, a consistent widespread cortical and subcortical involvement of gray and white matter affecting all lobes, brainstem and cerebellum was observed. Spectroscopy studies gave additional evidence of neuronal and glial damage in both types. Central questions regarding the origin and spatiotemporal evolution of the CNS involvement and its relevance for clinical symptoms had already been raised 30 years ago, however are still not answered. Results of correlation analyses between neuroimaging and clinical parameters are diverse and with few exceptions not well reproducible across studies. It may be related to the fact that most of the reported studies included only small numbers of subjects, sometimes even not separating Myotonic Dystrophy Type 1 from Type 2. But this heterogeneity may also support the current point of view that the clinical impairments are not simply linked to specific and regionally circumscribed structural or functional brain alterations. It seems more convincing that disturbed networks build the functional and structural substrate of clinical symptoms in these disorders as it is proposed in other neuropsychiatric diseases. Consecutively, structural and functional network analyses may provide additional information regarding the link between brain pathology and clinical symptoms. Up to now, only cross-sectional neuroimaging studies have been published. To analyze the temporal evolution of brain affection, longitudinal studies are urgently needed, and systematic natural history data would be useful to identify potential biomarkers for therapeutic studies.

## Introduction

Myotonic Dystrophy Type 1 (DM1) and Type 2 (DM2) are autosomal dominantly inherited multisystem disorders with overlapping clinical phenotypes. Structural and functional brain involvement is highly prevalent and clinically relevant in both disorders.

Various neuroradiological techniques have been applied over the years to examine the morphological structure as well as the function of the brain in DM1 and DM2. Methods range from conventional cranial computed tomography (CT) that were used in early days of brain imaging to advanced magnetic resonance imaging (MRI) techniques like functional MRI (fMRI). Various morphological MRI techniques have been applied to examine brain structure in myotonic dystrophies including conventional morphological MRI sequences, volumetric MRI methods (normalized brain and cortical volumes, respectively), brain parenchymal fraction (BPF), surface-based morphometry techniques (SBM) to analyze callosal body volumes, voxel-based morphometry (VBM) to analyze gray matter (GM), and white matter (WM) volumes/densities using voxel-based approaches, and diffusion MRI techniques (DTI) to examine WM microstructural integrity by voxel- or ROI-based approaches. Fluorodeoxyglucose positron emission tomography (FDG-PET) has been used to investigate the cerebral glucose metabolism in myotonic dystrophies. To analyze cerebral perfusion, ^99m^Tc-ECD and HMPAO single-photon emission computed tomography (SPECT) as well as H_2_O^15^-PET were applied. Analyses of cellular and neuronal markers have been performed by Proton-MR-spectroscopy (^1^H-MRS). Functional neuroimaging techniques were introduced to gain more insight into brain functioning and cerebral networks in myotonic dystrophies. Thus, functional MRI (fMRI) using e.g., motor tasks and resting-state fMRI have been applied in DM1 patients. Recently, transcranial B-mode sonography was used in adult-onset DM1 and DM2 patients for the first time to evaluate the echogenicity of brainstem and basal ganglia as well as ventricle diameters.

This review is based on studies reporting neuroimaging results in DM1 or DM2 patients applying at least one of the following methods: (functional) MRI, magnet resonance spectroscopy (MRS), SPECT, PET, or ultrasound. Search was done in Pubmed-database until March 1 2018, studies should be written in English and the full text of the study available. We also performed cross-referencing to identify articles potentially missed by our search.

### Myotonic dystrophy type 1

First cranial CT scans in DM1 had been conducted and published in the 80s and were the first real proof of a morphological brain affection in this disorder. These early neuroimaging data had given rise to a couple of scientific comments on clinical and radiological observations in DM1 that were published subsequently (1–3). DM1 patients showed increased ventricular surface areas and asymptomatic areas of focal cerebral atrophy (4). Further cranial CT studies gave evidence of microcephaly and thickening of the calvarium thus confirming cranial hyperostosis as a possible sign of brain pathology in DM1. Some patients also presented with basal ganglia calcification (5).

To our knowledge, the first publication on brain MRI in DM1 also included the first published cerebral CT image of one DM1 patient. The MRI study made use of a 0.5 T scanner for brain imaging. There was an increased incidence of ventriculomegaly in DM1 patients. Furthermore, this study gave first evidence of periventricular hyperintensities in DM1 patients when compared to controls (6).

First neuroimaging data in myotonic dystrophies included cranial CT of the brain and skull as well as 0.5 T brain MRI in congenital DM1, juvenile- as well as adult-onset patients and had been restricted to the type 1 form of the disease. In contrast, the first brain imaging study in DM2 was published only in 1997 (7). In general, CT imaging in those early days showed ventricular enlargement particularly in the congenital form of DM1, microcephaly, thickening of the calvarium, diffuse brain atrophy, cortical atrophy, and white matter hypodensities. Early reports on conventional morphological brain MRI findings in DM1 describe a wide variety of abnormalities ranging from dilated Virchow-Robin spaces, WM hyperintense lesions (WMHL) to cerebral atrophy(6, 8–12).

In the following years, several conventional structural brain MR studies were conducted in DM1 using MR scanners with field intensities of up to 1.5 T. The first “high-field” 3.0 T brain MRI studies however were performed in 2011 in congenital and juvenile-onset DM1 (13) and in adult-onset DM1 and DM2 patients (14). The latter study of Minnerop et al. included conventional structural brain MRI whereas the high-field study on congenital and juvenile-onset DM1 of Wodzniak et al. was focused on DTI techniques.

#### Conventional morphological brain MRI in DM1

Data base research for original articles published from 1988 to 2018 identified 40 systematic neuroimaging studies that included conventional structural/morphological MRI techniques in various series of DM1 patients using field intensities of 0.5–3.0 T (1, 6, 9–11, 14–46).

The numbers of studies using conventional, non-quantitative structural MRI techniques decreased in the recent years due to the implementation of more advanced and quantitative techniques like VBM and DTI as well as fMRI technologies.

The number of investigated patients by conventional MRI ranged from 2 to 60 patients per study, not respecting a review article on 66 DM1 patients and a very recent thorough review article on brain imaging in DM1 (23, 47). Most examinations focused on the classical adult-onset form of DM1, whereas few patient series included small numbers of congenital DM1 patients and juvenile-onset forms (6, 18, 21, 23, 26, 35).

Brain MR images were usually evaluated according to WM and GM structural abnormalities. Most studies were merely descriptive, especially in the beginning of MRI application in DM1, and not all studies compared patient data against healthy control subjects. Later, there have been strong attempts to quantify particularly the data on WM changes, and several scores to describe and quantify WMHL have been applied. WM lesions scores used in DM1 imaging studies included e.g., the age-related white-matter changes scale [ARWMC, (48)], and later also a visual scale according to a modified version of the Fazekas scale (43, 49). Due to the availability of WMHL scales and scores, the most uniform and comparable data of conventional morphological brain MRI are available for WMHL in DM1. In contrast, methods of GM evaluation are much more diverse and highly rater-dependent which might contribute to more controversial results on GM findings in DM1 conventional brain MRI studies. More advanced MRI techniques like VBM have largely replaced conventional brain MRI in DM1 to analyze GM, which allows a more rater-independent and quantitative analysis of GM abnormalities like cerebral atrophy in DM1.

#### White matter findings in DM1 using conventional morphological brain MRI

**Figure 1:**
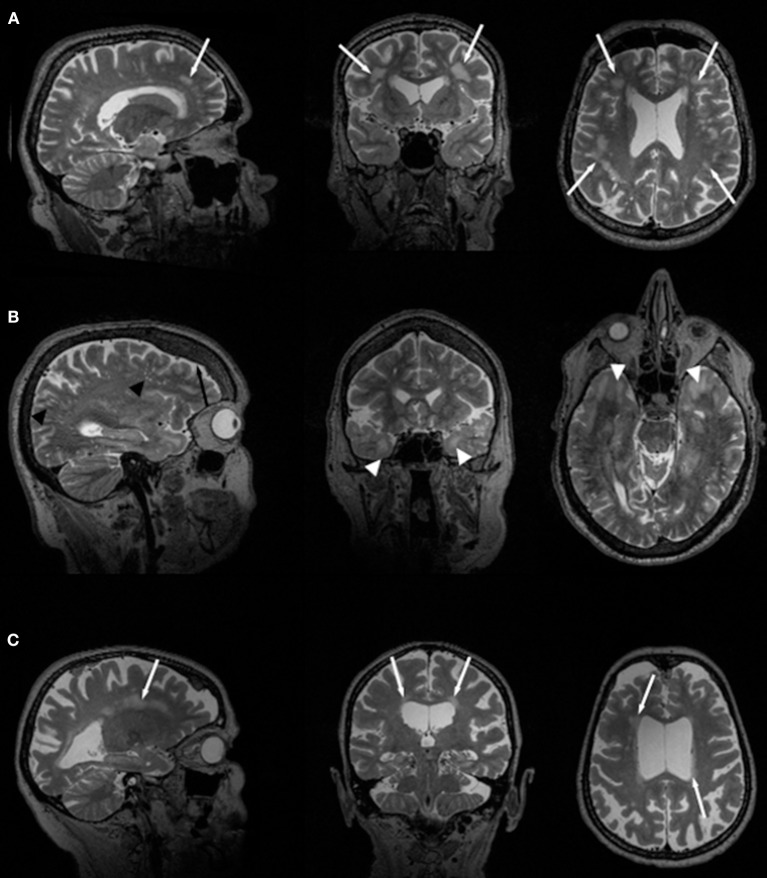
Slices of T2-weighted MR images in two different DM1 **(A, B)** and one DM2 **(C)** patients. Global atrophy with ventricle enlargement is seen in all cases. White arrows indicate WMHL, white arrow heads point toward ATWML. Black arrow indicates frontal hyperostosis in DM1 patients, black arrow heads point toward dilated Virchow-Robin spaces.

In general, WM changes and WMHL have frequently been reported in patients with DM1 (14, 24, 31). Also, thinning or atrophy of the callosal body has been reported most frequently in congenital DM1 but also in adult-onset forms of the disease (18, 19, 21, 46). WMHL in DM1 are predominantly located in frontal and temporal lobes (Figure 1A). Particularly, anterior temporal WML (ATWML) show a high prevalence in DM1 patients (Figure 1B). ATWML and a temporopolar WM pathology were identified in various and independent DM1 cohorts and constitute a highly robust finding over more than two decades of brain imaging. ATWML have been described early in DM1 (8) and could be reproduced only recently in brain MRI studies using more elaborated scanning techniques (43, 45, 46). ATWML seem to be a rather specific presentation of DM1 especially when compared to DM2 where this characteristic brain affection is usually not present (7–11, 14, 24, 31, 38).

The natural history of WM affection in DM1 is still a matter of debate and even conventional structural brain MRI data are controversial in this respect. Most cross-sectional analyses investigating the presence and extent of WMHL gave evidence of a potential progress over time or more pronounced WMHL in older patients when compared to children or adolescents with DM1, with age or disease duration used for correlation analyses. However, others did not find significant correlations with age or a significant increase of WML with disease progression (9, 11, 20, 23, 45, 46, 50, 51). In summary, longitudinal data on the presence and extent of WM abnormalities and WML in DM1 are scarce, and a systematic analysis of WM affection over time in DM1 patients compared against healthy controls by conventional MRI is still missing.

#### Gray matter findings in DM1 using conventional morphological brain MRI

In a recent review article, brain imaging studies on DM1 using magnetic resonance spectroscopy (MRS), fMRI, CT, ultrasound, PET, SPECT, but also conventional brain MRI has been analyzed. The authors had extracted data from a total of 81 studies on patients with DM1 (Embase, index period 1974–2016 and MEDLINE, index period 1946–2016). In conclusion, general brain atrophy and widespread GM volume reductions were reported in all cortical lobes, the basal ganglia, and cerebellum in DM1 (47).

Brain atrophy was described as a characteristic finding in DM1 very early. GM abnormalities reported in the literature by use of conventional morphological brain MRI techniques include ventricular enlargement, diffuse cortical atrophy, global GM reduction, focal brain atrophy in various cerebral lobes, the hippocampus, and basal ganglia. Cortical and subcortical GM atrophy is reported to be mostly symmetric and seems to be more pronounced in adult-onset compared to juvenile or congenital forms of DM1. Progressive GM loss in DM1 had been assumed earlier according to cross-sectional neuroimaging study results (14, 33, 38, 40, 44). However, there is discrepancy in the literature about the effect of including congenital DM1 patients on the extent of GM changes in neuroimaging analyses (38, 44). Some data hint to a more pronounced GM atrophy when congenital forms are included, others suggest the opposite. As in WM changes, the natural history of GM changes in DM1 is largely unclear, and longitudinal data on GM abnormalities are widely missing. To answer these still unsolved questions, systematic longitudinal brain imaging studies on large cohorts of congenital, juvenile- and adult-onset DM1 patients over time and against healthy controls would be needed.

### Structural brain imaging in DM1

The development of observer-independent neuroimaging techniques allowed quantifying brain atrophy, and specific properties of the brain could be accessed and analyzed by the use of specific MR sequences.

#### Quantification of global brain volume in DM1

Please see Table 1 for technical details of the included studies. In 2003, Kassubek et al. estimated brain atrophy in DM1 via brain parenchymal fraction (BPF) (52). This method comprises the segmentation of T1-weighted MR sequences into GM, WM and cerebrospinal fluid and the calculation of BPF by dividing the sum of GM and WM fractions by the sum of GM, WM and cerebrospinal fluid fractions - so far representing the total intracranial brain volume. Since BPF decreases during healthy aging and females have higher BPF values (53), age- and sex-matched control groups are required. In DM1, BPF values were markedly reduced, but did not correlate with clinical parameters like disease duration, motor score, educational level, or CTG repeat length.

**Table 1:** Quantification of brain volume in DM1.

**Study**	**Modality**	**Estimated parameters**	**Group size**	**Age (M ±SD or range) [years]**	**Disease duration (M ±SD or range) [years]**	**CTG repeat length (range)**	**Correlation parameters**
Kassubek et al. (52)	1.5T (T1)	BPF	10	36 ± 14	17 ± 10	110–1300	Disease duration, motor score, educational level, CTG
Antonini et al. (54)	1.5T (T1, T2)	GM/WM volume, TIV WMHL-load, VBM (GM)	22 (no congenital)	20–55	2–47	96–1570	Age, disease duration, MIRS, CTG, WMHL-load
Ota et al. (56)	1.0T (T1)	VBM (GM), corpus callosum volume	11 (no congenital)	56.6 ± 8.6	28.5 ± 15.5	+	Age, age at onset, disease duration, CTG (+)
Weber et al. (33)	1.5T (T1, T2, FLAIR)	BPF, VBM (GM)	14 (no con-genital/early-onset)	37.2 ± 14.2^*^	16.0 ± 9.6^*^	100–1300^*^	Disease duration (+), NPT (+), WMHL-load (+)
Minnerop et al. (11)	3T (T1)	VBM (GM, WM)	22 (no con-genital/childhood onset)	43.1 ± 12.6	13.2 ± 7.0	80–1100	-
Franc et al. (57)	3T (T1)	GM, ROI-based	10 (congenital +adult-onset)	24–34/30–43	-	+	FA (+)
Caso et al. (38)	1.5T (T1)	VBM (GM)	51 (17 juvenile)	42 ± 10	19.2 ± 8.5	177–1534	Age, disease duration, CTG, NPT, sleepiness
Schneider-Gold et al. (41)	3T (T1)	VBM (GM, WM), volumentry total GM/WM, cerebellum, brainstem, upper cervical cord, ventricle	12 (no con-genital/childhood-onset)	45 ± 13	18 ± 7	75–720	NPT (+), depression (+), daytime sleepiness, MIRS (+)
Serra et al. (40)	3T (T1)	VBM (GM)	10	41.8 ± 9.6	-	54–2000	CTG (+)
Baldanzi et al. (43)	3T (T1, FLAIR)	BPF, VBM (GM)	30 (only adult-onset)	44.6 ± 12.4	16.5 ± 11.8	E1, E2	Age, disease duration, NPT (+)
Zanigni et al. (44)	1.5T (T1, FLAIR)	VBM (GM), CT	24	38.5 ± 11.8	16.2 ± 10.8	E1, E2, E3	NPT, CTG, clinical scale
Cabada et al. (46)	1.5T (T1)	ROI- based (sub-) cortical GM-volume corpus callosum	40 (adult-onset)	37.3	-	+	Age (+), sleepiness (+), NPT (+)
Sugiyama et al. (58)	3T (T1)	VBM (GM), graph theory	28 (4 childhood, 10 juvenile)	42.5 ± 11.2	22.0 ± 12.4	133–3000	CTG, disease duration, age at onset, MIRS

All included studies investigated patients in comparison with controls. BPF, brain parenchymal fraction, CSF, cerebrospinal fluid; CT, cortical thickness; CTG, CTG repeat length; E1, 50–150 CTG repeats; E2, 150–1000 CTG repeats; E3, >1000 CTG repeats; GM, gray matter; MIRS, muscular impairment rating scale; NPT, neuropsychological tests; ROI, region of interest; TIV, total intracranial volume (GM+WM+CSF); VBM, voxel-based morphometry; WM, white matter; WMHL, white matter hyperintensity lesions; (+), positive or negative correlation was found;

* *data refer to whole DM1 study group, including patients that did not undergo MRI*.

Antonini et al. compared GM and WM volumes (not fractions) between non-congenital DM1 patients and controls and found significant reduced volumes in DM1 patients (54). The negative correlation of age with GM volume was stronger in patients than in controls, while WM volume did not correlate with age—neither in patients nor in controls. Similar to Kassubek et al. they found no correlation between brain tissue volumes and clinical parameters {disease duration, muscular impaired rating scale (MIRS, (55)), CTG repeat length, or WMHL load}. Antonini et al. stated correctly that their cross-sectional design did not allow differentiation between neurodevelopmental volume loss, acquired atrophy or progressive neurodegeneration, but postulated a neurodevelopmental GM loss which progresses with ageing because of the more pronounced negative correlation with age. The lack of any correlation between GM volume and WMHL-load led to the suggestion that WMHL and cortical atrophy progress as two independent processes.

Regarding BPF, Weber et al. confirmed previous findings from Kassubek et al. but described now a correlation with disease duration and WMHL load (33, 52).

Schneider-Gold et al. confirmed supratentorial brain atrophy in DM1, mainly driven by GM atrophy and accompanied by enlargement of all ventricular spaces, which also correlated negatively with each other (41). Supratentorial atrophy correlated also inverse with MIRS. However, the temporal horn index (temporal horn volume/lateral ventricle volume), supposed as an indirect and sensitive regional measure for (para-) hippocampal atrophy was only non-significantly elevated. No atrophy of the cerebellum or the upper cervical cord was detected.

Up to now, Baldanzi et al. analyzed the largest group of DM1 patients with respect to BPF and found a correlation with visuo-spatial and executive performance (43).

#### Quantification of regional brain volume in DM1

Please see Table 1 for technical details of the included studies.

Voxel-based morphometry (VBM) allows analyzing local volume changes within the brain and is based on an automated segmentation procedure of T1-weighted MR images into GM, WM and cerebrospinal fluid in combination with normalization to tissue-class specific templates. The procedures result in smoothed probability maps for each tissue class which can be compared between groups.

The first VBM study in non-congenital DM1 patients was performed by Antonini et al. demonstrating a widespread pattern of volume differences, located in frontal, parietal, temporal cortex bilaterally, and left superior occipital gyrus. Subcortical atrophy of the left caudate was detected as well (54). Subsequently published VBM studies in non-congenital DM1 patients largely confirmed a pattern of widespread cortical GM reduction affecting all lobes and frequently involving pre- and postcentral gyrus (14, 33, 38, 40, 41, 43, 44, 56, 58). Hippocampus atrophy was described by only few VBM studies (33, 38, 44, 58) and in one study even correlated with episodic memory (33). Subcortical changes are also part of the pattern observed in non-congenital DM1 patients and involve striatum, thalamus and cerebellum, albeit to a variable extent in different studies. Cabada et al. applied a methodological different, ROI-based approach to estimate GM volume and observed additionally subcortical reduced gray matter volume in the nucleus accumbens and ventral diencephalon (46).

Up to now, Zanigni et al. has performed the only analysis of cortical thickness in DM1 patients (44). Although VBM and cortical thickness analyses address both GM in T1-weighted MR images, there are methodological differences. VBM analysis provides a mixed measure of GM volume, including different structural properties, such as cortical thickness, surface area, and cortical folding (59). By combining both techniques, complementary information regarding brain structure can be obtained. Zanigni et al. found in line with the widespread GM involvement in VBM studies reduced thickness within lateral-occipital cortex bilaterally, right precentral and left superior-parietal, superior-temporal, and fusiform cortices (44).

Only a few studies analyzed WM changes with VBM in non-congenital DM1 patients (14, 41). Minnerop et al. observed WM changes encompassing the entire corpus callosum, fornices, cingulum bundle, and confluent WM reductions in every lobe (14). Subcortical WM changes were visible at pontine level, along middle cerebellar peduncles and within cerebellar WM. Schneider-Gold et al. described reduced WM volume in corpus callosum, thalamus, and WM adjacent to the pre- and post-central gyrus left-sided (41). Ota et al. and Cabada et al. confirmed with ROI-based approaches reduced volume of the corpus callosum (46, 56).

VBM studies comparing patient groups with different disease-onset are very limited. Franc et al. found reduced GM volumes only in adult-onset DM1 patients, but not in patients with congenital onset (57). Caso et al. compared juvenile and adult-onset DM1 patients and found in patients with juvenile form only small regions of bilateral cortical atrophy in the pre- and post-central gyri, SMA, orbitofrontal, dorsal frontal, and lateral temporal cortices, parietal regions, occipital cortices, left cingulate cortex, and right thalamus (38). Zanigni et al. observed after excluding patients with congenital/childhood-onset unchanged subcortical GM changes while the cortical GM reduction was less pronounced than for the entire group (44). Similar, restricting the cortical thickness analysis to patients without congenital/childhood-onset lead to more circumscribed altered cortical thickness, affecting only left fusiform, lingual, and inferior temporal gyri.

Seven studies investigated correlation of CTG repeat length with GM volume, but only two detected a correlation. Ota et al. found a negative correlation between CTG repeat length and GM volumes of the bilateral motor area and right prefrontal cortex (56). Serra et al. found significant correlations with GM volumes of cingulate gyri, orbitofrontal cortices, and frontal poles bilaterally and left pre-central gyrus (40). So if any correlations with CTG repeat length were detected at all, it was associated with motor-related and frontal areas.

Performing regression analysis, Schneider-Gold et al. revealed an association of pontine WM changes with depression score (41). Most neuropsychological parameters did not correlate with imaging parameters. Only flexibility of thinking correlated with GM volume within left medio-parietal cortex, belonging to the secondary visual cortex. Correlation analyses in the study of Baldanzi et al. revealed an association between delayed recall of verbal memory test and the volume of left postcentral, left middle, and inferior temporal gyri and left supramarginal gyrus (43).

Cabada et al. analyzed the volume of (sub-) cortical GM in a ROI-based approach (46). Compared with controls, there was an increase rate of cortex volume loss associated with age. Sleepiness was associated with volume loss in right pallidum and right ventral diencephalon. Visuospatial impairment was significantly correlated with ventricle enlargement and volume loss in part of the corpus callosum, bilateral cingulated isthmus, right lateral occipital, and pericalcarine cortex.

The most recent study by Sugiyama et al. applied for the first time graph theoretical analysis to investigate network metrics of a network between predefined brain regions based on GM in DM1 patients (58). In spite of pronounced GM volume reduction according to afore executed VBM analysis, measures of global and local network organization did not differ between DM1 patients and controls—probably due to compensatory mechanisms. The parameter betweenness centrality (BC) estimates the number of shortest paths that traverse a given node. High BC values imply that the respective node is a highly central “hub” for anatomical connections. Although the number of hubs was reduced in DM1 patients, there was an increased BC in left fusiform, superior temporal gyrus, superior frontal gyrus, and right precuneus, and a decreased BC in right caudate nucleus and putamen. The authors assumed that the increased BC in the left fusiform gyrus might be related to abnormalities of face perception in DM1 patients. The decreased BC in the striatum reflecting reduced structural connectivity may be correlated with schizotypal-paranoid traits, as it was discussed by Serra et al. for reduced functional connectivity in the striatum (36).

#### Quantification of white matter alterations in DM1

Please see Table 2 for technical details of the included studies.

**Table 2:** Quantification of white matter alterations in DM1.

**Study**	**Modality**	**Estimated parameters**	**Group size**	**Age (M ±SD) [years]**	**Disease duration (M ±SD) [years]**	**CTG repeat length (range)**	**Correlation parameters**
Di Constanzo et al. (62)	0.5T (T2-Relaxometry)	T2-relaxation time, ROI-based (GM, WM)	20	37.6 ± 13.8	11.8 ± 8.4	96–2930	WMHL, VRS (+), age (+), aget at onset, disease duration (+), VBR, CTG, MIRS
Naka et al. (60)	1.5T (T1, T2, FLAIR, MTI)	MTR, ROI-based, (NAWM, WMHL)	14	41.5 ± 10.5	14.3 ± 6.9	-	Disease duration (+), age, age at onset
Fukuda et al. (25)	1.5T DTI (d6, b500)	ROI-based (FA, MD) (NAWM, WMHL)	19	43.9 ± 10.9	13.7 ± 9.0	-	Age, age at onset, disease duration
Ota et al. (56)	1.0T DTI (d12, b700)	ROI-based (FA, MD) tractography of corpus callosum	11 (no congenital)	56.6 ± 8.6	28.5 ± 15.5	+	Age, age at onset, disease duration, CTG
Wozniak et al. (13)	3T DTI (d30, b1000)	ROI-based (FA, MD, AD, RD)	8 (congenital + juvenile onset)	13.8 ± 2.3	-	200–1700	NPT (+)
Minnerop et al. (11)	3T DTI (d30, b1000)	DTI-TBSS (FA, MD, RD, AD)	22 (no congenital/childhood onset)	43.1 ± 12.6	13.2 ± 7.0	80–1100	Age (+), disease duration (+), depression (+), fatigue (+), NPT CTG (+), MIRS (+)
Franc et al. (57)	3T DTI (d12, b1000)	ROI-based (FA)	10 (congenital + adult- onset)	29.5/38.3	-	+	GM (+)
Wozniak et al. (61)	3T DTI (d30, b1000)	probabilistic tractography, ROI- based (FA, MD, RD, AD)	16 (congenital, childhood/juvenile onset)	13.9 ± 3.0	-	(+)	NPT (+)
Wozniak et al. (37)	3T DTI (d30, b1000)	probabilistic tractography, ROI- based (FA, MD)	45 (juvenile/adult-onset)	38.4 ± 6.6	-	75–800	NPT (+), MIRS (+), CTG (+), sleepiness (+)
Caso et al. (38)	1.5T DTI (d65, b1000)	DTI-TBSS (FA, MD, RD, AD)	51 (17 juvenile)	42 ± 10	19.2 ± 8.5	177–1534	Age, disease duration, CTG, NPT (+), sleepiness, WMHL-load
Serra et al. (40)	3T DTI (d61, b1000)	DTI-TBSS (FA)	10	41.8 ± 9.6	-	54–2000	CTG (+), NPT (+), MIRS (+)
Baldanzi et al. (43)	3T DTI (d25, b1000)	DTI-TBSS (FA, MD, RD, AD)	30 (adult-onset)	44.6 ± 12.4	16.5 ± 11.8	E1, E2	Age, disease duration, NPT (+)
Zanigni et al. (44)	1.5T DTI (d25, b900)	DTI-TBSS (FA, MD, RD, AD)	24	38.5 ± 11.8	16.2 ± 10.8	E1, E2, E3	NPT (+), CTG, clinical scale (+)
Cabada et al. (46)	1.5T DTI (d30, b1000)	DTI-TBSS (FA, MD, RD, AD)	40 (adult-onset)	37.3	-	+	Age, NPT (+), sleepiness, WMHL-load (+)

*All included studies investigated patients in comparison with controls. AD, axial diffusivity; b, b- factor [s/mm^2^]; CTG, CTG repeat length; d, number of directions of diffusion-encoding gradients; DTI, diffusion tensor imaging; E1, 50–150 CTG repeats; E2, 150–1000 CTG repeats; E3, >1000 CTG repeats; FA, fractional anisotropy; MD, mean diffusivity; MIRS, muscular impairment rating scale; MTI, magnetization transfer imaging; NAWM, Normal appearing white matter; NPT, neuropsychological tests; RD, radial diffusivity; ROI, region of interest; TBSS, tract-based spatial statistics; VBR, ventricle-to-brain ratio; VRS, Virchow-Robin-spaces; WMHL, white matter hyperintensive lesions; (+), positive or negative correlation was found*.

In the last two decades an increasing number of advanced MR methods were developed to specifically analyze structural changes within WM.

Di Constanzo et al. performed the currently only study in adult-onset DM1 patients applying T2 relaxometry (62). This method has been shown to be useful in quantifying signal changes on T2-weighted images, on which also in DM1 characteristic abnormalities are frequently observed. Relaxation times estimated in normal-appearing white matter (NAWM) and (sub-) cortical regions (striatum, thalamus) were prolonged in particular in WM. Total WM T2 values correlated with age and disease duration, while no correlations were found for specific WM regions or for GM. The detected changes within NAWM had not been reported before and pointed for the first time toward a more diffuse involvement of WM in DM1. The correlations with age and disease duration, but not with CTG repeat length were interpreted as possible signs of progressive changes in disease course.

Naka et al. used magnetization transfer imaging to analyze WM in DM1 patients (60). This method is based on the interaction between (myelin-) bound and free protons and is frequently applied in demyelinating disorders. Magnetization transfer is induced by an MR pulse saturating bound protons and differences of the signal intensity before and after inducing Magnetization transfer are measured. The authors found, consistent with the relaxometry–study, reduced MT ratios in WHML more than in NAWM, and in both a correlation with disease duration was observed.

The first diffusion tensor imaging (DTI) study in DM1 patients was performed by Fukuda et al. (25). DTI evaluates the magnitude and directionality of the diffusion of water molecules. The biophysical background of diffusivity changes in WM is still not fully understood. Next to myelin changes other structural abnormalities (e.g., axonal membranes) may play role. Within WM, diffusion is usually anisotropic (“directed”) due to the structural constraints of fiber tracts. Pathological disturbances of these microstructural barriers alter the diffusion behavior of water molecules, and reduce for example the anisotropy of diffusion within WM. This anisotropic diffusion can be quantified with the parameter fractional anisotropy (FA) in which higher values indicate more anisotropic, directed diffusion. Next to FA other diffusivity parameters exist: Like FA, the parameters radial and axial diffusivity (RD, AD) are derived from the same mathematical tensor model underlying DTI. RD and AD are thought to define the amount of diffusivity in the direction (axial) and perpendicular (radial) to the direction of fiber tracts. In this respect, radial diffusivity is thought to reflect alterations of myelin or axonal membranes while axial diffusivity may reflect pathological changes of the axon itself. Mean diffusivity (MD) measures the general diffusivity without paying attention to the direction of the diffusivity.

Fukuda et al. (25) compared similar to Naka et al. (60) and Di Constanzo et al. (62) method-specific measures within WMHL and NAWM applying a ROI-based approach. And again, they found lower FA and higher MD values in both, WMHL and NAWM, but more pronounced in WMHL. But in contrast to Di Constanzo et al. (62) and Naka et al., they did not find any correlation with disease duration or any other clinical parameter and assumed a non-progressive character of changes within NAWM (25, 60, 62).

Ota et al. showed diffusivity abnormalities within the corpus callosum for the first time in non-congenital DM1 patients and linked them to reduced cortical GM volume in different lobes (56). Not affected were only parietal lobes and the isthmus of the corpus callosum, both connected by fiber tracts. Furthermore, CTG repeat length correlated with cortical GM loss – but not with diffusivity parameters within the corpus callosum. The authors postulated Wallerian degeneration as underlying pathology of callosal diffusivity changes.

In our own study (Minnerop et al.) we confirmed changes of diffusivity within the corpus callosum in adult-onset DM1 patients (sparing only parts of the splenium), but found in a voxel-based approach extensive and wide-spread abnormalities throughout the entire brain (14). This involved association fibers, limbic system fiber tracts and projection fibers within internal and external capsules, including also changes of the corticospinal tract within the posterior limb of internal capsules and at brainstem level. Reduced FA was mainly accompanied by increased MD and RD, but increased AD was also present. Subsequent DTI studies confirmed this pattern of widespread diffusivity abnormalities (38, 40, 43, 44, 46).

Congenital and juvenile-onset DM1 patients were shown to have as well generalized changes of diffusivity parameters (reduced FA, higher MD, AD, and RD) and this was later also confirmed for children and adolescents with DM1 applying a tractography approach (13, 61). Franc et al. used the same set of large ROIs as in Wozniak et al. (13) to investigate congenital and adult-onset DM1 patients and found reduced FA values in both groups with lower values for the adult-onset DM1 group (57). Caso et al. included the up to now largest group of DM1 patients into a multimodal imaging study, allowing even to compare the juvenile with the adult-onset form (38). Results with respect to diffusivity parameters were similar when adult-onset and juvenile-onset DM1 patients were compared separately to controls. Zanigni et al. confirmed again the widespread pattern of microstructural damage in DM1 patients, independently if patients with very long CTG repeats were included or not (44).

Only three out of seven studies investigating correlations between CTG repeat length and diffusivity parameters found a correlation. Wozniak et al. reported in their tractography-based study of juvenile/adult-onset DM1 patients an association of CTG repeat length with MD values of the corticospinal tract and cingulum (37). Minnerop et al. described in adult-onset DM1 patients correlations with FA values of association fibers, fornix, cingulum bundles, corpus callosum, and left external capsule (14). Serra et al. described a correlation with FA values across the entire brain, including corpus callosum, brainstem and cerebellum (40). Zanigni et al. found only a correlation, as long as patients with very long repeats were not excluded (44).

Associations between cognitive performance and diffusivity parameters were investigated in nine studies and at least some kind of correlation was reported in seven. In congenital and juvenile-onset DM1 patients, Wozniak et al. found a correlation for whole brain FA values with lower IQ and executive functioning (13) and a ROI-based analysis in childhood/juvenile-onset DM1 patients revealed an association between FA and MD values (in particular of frontal and temporal lobes) with working memory performance (61). Several studies showed in non-congenital DM1 patients correlations between diffusivity parameters and neuropsychological test results. Serra et al. found correlations of generalized FA reductions with MMSE scores (40). Zanigni et al. found correlations between the MMSE score and diffusivity changes within the splenium and the posterior part of the corpus callosum, posterior corona radiate, and posterior thalamic radiations bilaterally and right retrolenticular part of the internal capsule (44).

Cabada et al. found correlations between MD and FA values of the posterior corpus callosum and visuospatial impairment (46). Similar, Baldanzi et al. observed an association between RD and AD values of the corpus callosum and visuomotor coordination and working memory tasks (43). Further associations were detected between visuo-spatial and episodic verbal memory and associative tracts of the internal capsule and coronal radiata. Caso et al. found a correlation between MD values of the left corona radiata and association fibers within the frontotemporal WM regions and orientation and attention scores (38). Working memory performance was correlated with MD values of all tracts investigated by Wozniak et al. while processing speed was associated with MD values of the corticospinal tract and association fibers (37).

Correlations with the MIRS score were found in three studies (14, 37, 40). Correlations with disease duration or age were investigated by five (disease duration), respectively seven (age), DTI studies, but a correlation between FA values and disease duration or age was only found by one (14). Noticeably, the pattern of affected structures was identical between correlation analysis of age and disease duration. Associations between diffusivity parameters and sleepiness or fatigue were investigated in four studies, but only two found significant correlations (14, 37). FA values in Minnerop et al. were lower in patients with less fatigue and less depressed mood (14). This somewhat contradictory finding of higher FA values associated with more depressed mood was interpreted in line with the work by Winblad et al. (63) as possible hint toward reactive depressed mood in early disease stages and the presence of more effective coping strategies or less abilities to perceive own limitations in later disease stages (associated with lower FA values). Fatigue correlated only minor with FA values of the corpus callosum, but showed instead a correlation with FA values of the brainstem (14). Wozniak et al. found MD values of association fibers related to sleepiness (37).

### Functional brain imaging in DM1

#### Positron emission tomography (PET) and single photon emission computed tomography (SPECT) in DM1

Please see Table 3 for technical details of the included studies.

**Table 3:** Positron emission tomography (PET) and Single photon emission computed tomography (SPECT) in DM1.

**Study**	**Modality (Tracer)**	**Estimated parameters**	**Group size**	**Age (M ±SD) [years]**	**Control group**	**Disease duration (M ±SD) [years]**	**CTG repeat length (range)**	**Correlation parameters**
Fiorelli et al. (15)	PET (^18^FDG)	glucose consumption	11	35.3 ± 11.2	+	-	-	Cortical atrophy
Mielke et al. (64)	PET (^18^FDG)	glucose consumption	3	42/50/59	+	-	-	-
Chang et al. (16)	SPECT (^133^Xe, ^99m^TC-HMPAO)	CBF, perfusion	22 (no congenital)	36.6 ± 14.0	+	13.5 ± 6.9	-	NPT (+)
Annane et al. (65)	PET (^18^FDG)	glucose consumption	11	43 ± 12	+		250–5000	CTG (+), plasma insulin level (+)
Meola et al. (42)	PET (H_2_O^15^)	CBF	11	42.7 ± 14.6^*^	+		500–700^*^	-
Takeda et al. (66)	SPECT	CBF	2	35/55	-	25/19	1300	-
Romeo et al. (69)	SPECT (^99m^TC-ECD/HMPAO) PET (^18^FDG)	perfusion/glucose consumption	58 (+PET: 17)	46 ± 15/+PET: 49 ± 9	-	12.2 ± 8.6	+	MIRS (+)
Weber et al. (33)	PET (^18^FDG)	glucose consumption	17 (no con-genital/early-onset)	37.2 ± 14.2^*^	+	16.0 ± 9.6^*^	-	NPT
Renard et al. (67)	PET (^18^FDG)	glucose consumption	24 (no congenital)	47 ± 12.5	+	19.1 ± 9.0	83–2000	CTG, age at onset
Peric et al. (68)	PET (^18^FDG)	glucose consumption	16 (no con-genital/late-onset)	45.6 ± 9.6	+	21.8 ± 8.3	-	NPT (+)

CBF, cerebral blood flow; CTG, CTG repeat length; ^18^FDG, ^18^F-Fluordesoxyglucose; MIRS, muscular impairment rating scale; NPT, neuropsychological tests; PET, positron emission tomography; SPECT, single photon emission computed tomography; (+), positive or negative correlation was found;

* *data refer to whole DM1 study group, including patients that did not undergo PET*.

The first functional brain imaging study on adult DM1 patients was performed by Fiorelli et al. in 1992 using FDG-PET (15). They found about 20% reduced glucose utilization rate in DM1 patients in comparison to controls. Mielke et al. found reduced uptake in all cortical and subcortical regions, predominantly in frontal regions and lentiform nucleus (64), and Annane et al. confirmed these results and reported a negative correlation between glucose consumption and CTG repeat length and plasma insulin levels, reflecting peripheral insulin resistance (65). Renard et al. reported—albeit not corrected for partial volume correction—bilateral symmetrical reduced FDG uptake in the lateral part of the frontal lobes, affecting most severely Brodmann area 8 (related to eye movement control), while deep GM structures did not show reduced metabolism (67). CTG repeat length did not correlate with hyopmetabolism, although there was a tendency toward lower FDG uptake with CTG repeat length >1000 and early (childhood) onset DM1, congenital cases were not included. Weber et al. also reported widespread and symmetric hypometabolism in the frontal lobes stretching to part of the temporal lobes (33). This finding cannot simply be attributed to cortical atrophy, since results remained unchanged even after partial volume correction in a subgroup. However, no correlations with NPT were found. The most recent FDG-PET by Peric et al. analyzed glucose metabolism and its relation to neuropsychological testing, excluding congenital and late-onset DM1 cases (68). The most prominent hypometabolism was present in prefrontal, frontotemporal, temporal, and precentral regions, subcortical GM regions were less affected than cortical areas. Right frontotemporal hypometabolism correlated with executive dysfunction.

Chang et al. investigated cerebral blood flow and perfusion via SPECT ligands and compared subgroup of DM1 patients with maternal and paternal inheritance (16). Although no congenital cases were included, according to the demographic data at least subjects with childhood onset were included. Blood flow (CBF) and perfusion was reduced compared to controls, particular in the temporal and frontal regions and regional CBF correlated with NPT performances, while the mode of inheritance had significant impact only on perfusion, but not on CBF—most likely due to low resolution of the ^133^Xe-SPECT measuring CBF. Meola et al. described a marked reduction CBF via H_2_O^15^-PET in orbitofrontal, medial and dorsolateral frontal cortex, temporal pole, hypothalamus and left basal ganglia (22). Reduced temporal and frontal CBF was also reported in two DM1 cases (one with childhood onset) (66). Romeo et al. analyzed retrospectively perfusion (SPECT) in a large group of DM1 patients—but without matched controls (69). A subgroup of patients also received FDG-PET. They described global hypoperfusion (and to a lesser extent reduced glucose metabolism) more pronounced on the left hemisphere, frontal regions, and general in cortical regions more present than in subcortical structures. With respect to cortical regions they also described a hypoperfusion gradient from frontal, parietal, temporal, occipital sensory-motor to insular regions, and frontal hypoperfusion even correlated with MIRS scores.

#### Proton MR spectroscopy (^1^H-MRS) in DM1

Please see Table 4 for technical details of the included studies.

**Table 4:** Functional brain imaging in DM1.

**Study**	**Modality**	**Estimated parameters**	**Group size**	**Age (M ±SD) [years]**	**Disease duration (M ±SD) [years]**	**CTG repeat length (range or M ±SD)**	**Correlation parameters**
Hashimoto et al. (79)	1.5T ^1^H-MRS (STEAM) TE 270 ms	ROI: parietal (NAA/Cho, NAA/Cr, Cho/Cr)	5 (congenital)	7.3 ± 5.5	congenital	-	Age (+)
Chang et al. (70)	1.5T ^1^H-MRS (PRESS) TE 30 ms	ROI: midoccipital GM + temporo-parietal GM left NAA, Cr Cho, myoinositol	14	37.8 ± 2.7	13.8 ± 3.5	173–1434	CTG (+), NPT
Akiguchi et al. (71)	1.5T ^1^H-MRS (STEAM) TE 19 ms	ROI: Insula (NAA/Cho, NAA/Cr, Cho/Cr)	21 (no congenital)	37.0 ± 13.6	11.2 ± 7.6	(+)	Age
Vielhaber et al. (72)	1.5T ^1^H-MRS (PRESS) TE 135 ms	ROI: frontal WM + midoccipital GM + temporo-parietal NAA, Cho, Cr	14 (no congenital/juvenile onset)	38.8 ± 9.1	-	250–750	Age, age at onset, disease duration, NPT, CTG
Takado et al. (73)	3T ^1^H-MRS ^1^H-MRSI (PRESS) TE 30 ms, 144 ms	ROI: ant. cingulate gyrus, frontal WM, Slices BG level, (tNAA, tCho, Cr, MI, Glu, Gln, Glx, NAA/Cho, NAA/Cr, Cho/Cr)	13	43.6 ± 12.6	11.8 ± 9.0	685 ± 462	NPT (+), CTG (+)
Caramia et al. (32)	1.5T fMRI	self-paced sequential finger-to-thumb opposition task (right hand)	15	36.3 ± 12.3	15.4 ± 12	96–1570	Age (+), disease duration, MIRS score, WMHL-load
Toth et al. (74)	3T fMRI	myotonia-inducing grip task prior and after warm-up procedure	16	47.9 ± 8.0	18.3 ± 9.0	+	-
Serra et al. (36)	3T rsfMRI	DMN functional connectivity	27 (no congenital)	39 ± 11.8	-	54–2000	Personality traits/disorders (+)
Serra et al. (80)	3T rsfMRI	theory of mind-network functional connectivity/graph theory	20 (no congenital)	43.9 ± 10.7	-	150–1200	-
Serra et al. (82)	3T rsfMRI	functional connectivity/graph theory	31 (no congenital)	39.9 ± 11.4	-	54–2000	MIRS (+), CTG, NPT (+)
Park et al. (75)	3T rsfMRI	sensorimotor network functional connectivity/power spectral density	18 (adult-onset)	44.4 ± 10.7	13.4 ± 2.0	374 ± 66	Motor performance (+), CTG (+), disease duration (+)
Krogias et al. (76)	ultrasound	echogenicity of basal ganglia/mesencephalic regions, ventricle diameters	17	39 ± 15	17 ± 6	75–1000	Age (+), CTG (+), daytime sleepiness (+), depression
Peric et al. (77)	ultrasound	echogenicity of basal ganglia/mesencephalic regions, ventricle diameters	61 (no congenital)	41.2 ± 10.3	18.9 ± 8.6	747 ± 280	Sex (+), disease duration (+), CTG, MIRS, depression (+) fatigue (+), RLS

*All included studies investigated patients in comparison with controls. Cho, choline; Cr, creatine; CTG, CTG repeat length; DMN = default mode network; fMRI, functional MRI; Gln, Glutamin; Glu, Glutamate; Glx, Glu+Gln; GM, gray matter; MI, myo-inositol; MIRS, Muscular Impairment Rating Scale; MRS, magnetic resonance spectroscopy; NAA, N-acetyl aspartate; NPT, neuropsychological tests; PRESS, point resolved spectroscopy (MR pulse sequence); ROI, region of interest; rsfMRI, resting state functional MRI; SE, spin echo technique; STEAM, stimulated echo acquiring method (MR pulse sequence); tCho, glycerophosphcholine + phosphocholine; tNAA, NAA + N-Acetylaspartylglutamate; WMHL, white matter hyperintensive lesions; (+), positive or negative correlation was found*.

^1^H-MRS allows analyzing biochemical properties of the brain *in vivo*. Metabolites commonly measured are N-acetylaspartate (NAA), phosphocreatine (Cr), and choline (Cho). NAA is only present in neurons (neuronal marker), and neuronal loss is usually correlated with decrease of the NAA content. Cho represents a membrane-bound molecule of neuroglial cells and can be regarded as glial marker. Creatine is ubiquitously expressed in brain and reflects the energy potential available in brain tissue. Measuring these metabolites at longer echo time is advantageous regarding signal-to noise ratio: Interference with signals from other metabolites is avoided since their peaks occur at shorter TE and have already decayed. Contrary, applying short TE allows to quantify a spectrum of additional neurochemical alterations of amino acids relevant to neural transmission, cell structure, and cell energy metabolism, e.g., glutamine, glutamate, or myo-inositol, a naturally occurring sugar regarded as an glial-specific marker.

Concentrations of specific metabolites can be measured either by single-voxel spectroscopy, where a single sample volume is analyzed, or multi-voxel spectroscopy where multiple voxels in a single slab of tissue are analyzed (MR spectroscopy imaging). Since estimating absolute NAA levels *in vivo* are difficult to obtain and to avoid bias due to interindividual differences, the content is often measured in relation to other molecules (Cho, Cr), but absolute values are advantageous [for methodological details see (78)].

The first study applying ^1^H-MRS in five children with congenital DM1 was performed in 1995 by Hashimoto et al. (79). ROIs were placed within right parietal region and in some cases additional ROIs were placed in occipital and frontal regions. Occipital/parietal regions partially included areas with WMHL in two cases. While in matched controls an age-dependent increase of NAA/Cho and NAA/Cr was observed, the NAA/Cho ratio did not correlate with age in DM1 patients and the NAA/Cr on the contrary decreased with age. The Cho/Cr ratio did not differ, showing a decrease with increasing age in both groups. The authors concluded that the decreases in NAA/Cho and NAA/Cr were therefore most likely caused by a decrease of NAA, pointing toward neuronal damage or defective development. A decrease of NAA/Cho and NAA/Cr ratios were found independently of the presence of WMHL in the respective ROI.

Chang et al. reported in a mixed patient group including congenital, juvenile and adult-onset DM1 patients elevated levels of myo-inositol, total creatine, and choline-containing compounds, pointing to an increased glial content in the two brain regions (occipital region, temporoparietal region) studied (70). NAA levels did not differ between DM1 patients and controls. Furthermore, the creatine and myo-inositol peak areas correlated with the CTG repeat length, especially in the temporoparietal brain region.

Akiguchi et al. analyzed in non-congenital DM1 patients NAA/Cho and NAA/Cr in an insular ROI (71). Both ratios were reduced while Cho/Cr did not differ. None of the ratios correlated with age and no difference between patients with and without a subnormal MMSE scores was found. Without measuring absolute values, it remained unclear whether the abnormal ratios were due to reduced NAA (pointing toward neuronal damage) or due to increased Cho or Cr (pointing to increased glial content). But in the light of the study by Chang et al. the authors assumed that their results were reflecting also increased glial content.

Vielhaber et al. estimated in adult-onset DM1 patients absolute values of NAA, Cr and Cho as well as ratios in three ROIs located within midoccipital and temporoparietal GM, but also in frontal WM (72). In contrast to Chang et al. (70), NAA was reduced in all three ROIs and most pronounced in frontal WM, while Cr and Cho were reduced in temporoparietal GM and to a less extent also in the frontal WM. Accordingly, NAA/Cr and NAA/Cho ratios were reduced (13–21 %) while Cho/Cr ratio remained unchanged compared to controls. However, no MRS-parameter correlated with clinical parameters. The authors concluded that glial loss or dysfunction could be a possible cause of the Cr and Cho depletion, while neuronal loss most likely caused the NAA loss. They further postulated that the lack of correlation with age of onset or disease duration argues against primary neurodevelopmental or progressive process in adult-onset DM1.

Takado et al. applied single voxel ^1^H-MRS within regions of the frontal WM and the anterior cingulate gyrus and MRSI investigating two slices at the basal ganglia or upper lateral ventricles level (73). By MRSI they found decreased NAA/Cr ratio in insula cortex, putamen, thalamus, internal capsule (posterior limb), frontal, and posterior WM. Cho/Cr ratio was increased in the thalamus and reduced in FWM. Similar, single-voxel ^1^H-MRS showed decreased NAA and NAA/Cr in both ROIs (anterior cingulate gyrus and frontal WM), Cho and glutamine (in frontal WM). After correcting for partial volume effects a significant increase in Cho, glutamine, and glutamate concentrations were found in the anterior cingulate gyrus, pointing toward disturbed glutamatergic system with the frontal lobe. NAA/Cr in frontal WM correlated with NPT measures and with the CTG repeat length. The authors concluded that neuronal abnormalities seem to occur, both, in GM and WM, without significant gliosis.

#### fMRI and resting-state fMRI in DM1

Please see Table 4 for technical details of the included studies.

Caramia et al. investigated a self-paced sequential finger-to-thumb opposition task (right hand) and found a greater activation in DM1 patients compared to controls in bilateral sensorimotor areas, inferior parietal lobules, basal ganglia and thalami and ipsilateral premotor area, insula and SMA (32). Further, age correlation was greater in patients than in controls in bilateral sensorimotor areas and in contralateral parietal areas. The authors hypothesized that the increased brain motor activation reflects a compensatory mechanism and because of similarities with alterations found in healthy aging. They postulated that the observed changes are part of an accelerated aging process.

While Caramia et al. (32) tried to avoid any interference of their fMRI-motor task with myotonia, Toth et al. specifically investigated the impact of myotonia on cerebral functioning via fMRI (74). They compared DM1 patients with and without grip myotonia while performing a grip task before and after a warm-up procedure. In patients presenting grip myotonia they found higher activity within the SMA and the dorsal anterior cingulate cortex (ACC). The same regions were also activated when fMRI activity was compared before and after the warm-up session in patients with grip myotonia. No activation of primary motor areas occurred during myotonia, which was interpreted by the authors as further evidence against an involvement of brain function in the development of myotonia. Instead, the observed activity probably reflects compensational cortical activations. SMA is part of active inhibitory circuits which are usually activated preceding voluntary muscle relaxation, so the authors postulated that activation within this region during myotonia may reflect the unconscious intention to finish the abnormally prolonged muscular activation. In turn, ACC links cognitive functions to motor actions and seems to play a role in error detection. So the authors hypothesized that the increased activity within the ACC could be related to the error-detection of experiencing non-occurring grip relaxation despite the intention to do so.

Instead of measuring the BOLD (blood oxygen level—dependent) signal in response to specific task as mentioned above, resting state fMRI is a functional MRI technique investigating fluctuation of the BOLD signal of the brain in rest without performing an active task. Functional connectivity can be estimated between spatially distinct brain regions by identifying temporal synchronous frequency fluctuations between the respective regions. This leads to the identification of specific function- or disease- related networks. One of the most frequently investigated networks is the default mode network (DMN) comprising brain regions which are active in wakeful rest and deactivated when performing active tasks.

Serra et al. investigated for the first time the interaction of personality traits/disorders in DM1 patients and functional connectivity within the DMN (36). Functional connectivity was increased within the bilateral posterior cingulate and left parietal DMN nodes. Additionally, DMN functional connectivity within the left supramarginal gyrus (parietal node) and right putamen and caudate nucleus (inverse correlation) was strongly associated with schizotypal-paranoid traits in DM1 patients. The authors postulated that an overengagement of the DMN may lead to an exaggerated focus on one's own thoughts and feelings. The altered functional connectivity of the basal ganglia might be related to reduced cognitive flexibility in association with schizotypic—paranoid traits, probably accounting for patients rigid thoughts and fixed ideas.

In a consecutive study by the same group (Serra et al.), the authors investigated social cognition with the Theory of Mind (ToM) framework in relation to resting-state functional connectivity (80). The authors applied Graph theory to analyze functional networks. Graph theory-based approaches model the brain as a complex network represented graphically by a collection of nodes, indicating anatomical elements (e.g., brain regions) and edges (e.g., connectivity between nodes). After generating a network model, several metrics can be used to characterize and even quantify network properties on a local and global level (81). Some nodes are more critical (i.e. centrality) for information processing (efficiency in information transferring) and are called “hubs”. Serra et al. identified a ToM network containing 14 nodes and 9 edges (80). While global topological properties of the identified ToM network did not differ between DM1 patients and health controls, comparison of local properties showed a significant increase of nodal efficiency and degree in the left inferior temporal gyrus. In DM1 patients, this region was significantly more connected to dorsolateral prefrontal cortex and cerebellum than in controls. In contrast, connections between inferior temporal gyrus and occipital regions were only observed in healthy subjects. The observed deficits within ToM tests in DM1 patients in association with abnormal connectivity between the left inferior temporal and fronto-cerebellar nodes, further underpins that difficulties in social interactions as well as personality traits are related to brain abnormalities and should not be regarded as reactive symptoms.

In another study by Serra et al. resting state fMRI data were analyzed with network-based analysis and graph theory (82). Additionally, correlation analysis between network metrics and clinical data were performed. Dysfunctional hubs were located in the bilateral anterior cingulum, orbitofrontal cortex, and right parahippocampal gyrus. Connectivity correlated with NPT (visuospatial reasoning). With respect to graph theory analysis, no global, but local measures of connectivity differed significantly between groups (nodal degree, betweenness centrality, nodal efficiency). The anterior pattern with decreased fronto-parietal connectivity could probably be linked to cognitive and behavioral symptoms in DM1 patients. The posterior pattern with increase connectivity in SMA and cerebellum resembles according to the authors to pattern observed in patients with autism spectrum disorders and could represent repetitive stereotyped behaviors observed in autism and autism-like traits have been reported it patients with congenital DM1. Additionally, the regions involved in the posterior pattern are motor-related regions and may be relevant for the motor impairment in DM1 patients, The authors propose that the abnormally high connectivity in these motor regions might represent a compensatory, albeit inefficient or maladaptive mechanism of brain plasticity.

The most recent functional MRI study by Park et al. investigated power spectral density (PSD) in the resting-state sensorimotor network in DM1 patients (75). In contrast to functional connectivity analysis based on the correlation analysis between different brain regions, they applied power spectral density (PSD) analysis. Power spectrum is a physical quantity that can quantitatively reflect energy density (and consumption) changes, e.g., of low-frequency BOLD fluctuations. Compared to controls there was in DM1 patients a decrease of PSD in right superior temporal pole, and bilaterally in middle and inferior temporal gyrus, postcentral gyrus, occipital gyrus, precuneus, posterior cingulate, and cerebellum. An increase of PSD was detected in the orbitofrontal cortex, putamen, parahippocampal gyrus, fusiform gyrus, anterior insula cortex, and pallidum. Higher PDS responses were also found in WM structures (cerebral peduncle, head of caudate nucleus, anterior/posterior limb of internal capsule, externa capsule and cortical association fibers). Correlation analysis revealed an association between brain regions with altered PSD and motor performance, CTG repeat length, and disease duration. The authors concluded that motor disability in DM1 maybe strongly associated with abnormality in the visual processing network and that GM and WM PSD alterations seem to be involved in motor deficits in DM1 patients.

#### Transcranial ultrasound in DM1

Please see Table 4 for technical details of the included studies.

Transcranial B-Mode sonography is well-established in neonatology and has been used in congenital DM1 early to assess brain structure and integrity. Intracranial pathological findings like intracerebral hemorrhage, hydrocephalus or ventricular enlargement can be easily and non-invasively detected by application of this bedside technology. In congenital DM1, cerebral ventricular enlargement has frequently been described in single cases or smaller series of patients (83–85).

Only in recent years, the technique of transcranial sonography has been applied to adult-onset DM1 patients to assess the echogenicity of the brainstem raphe, mesencephalon and substantia nigra as well as the third ventricle width in cross-sectional study designs (76, 77). The authors reported that brainstem raphe hypoechogenicity was more common in DM1 patients than in controls, and both hypoechogenicity and hyperechogenicity of the substantia nigra were more frequent in DM1 patients. Moreover, the width of the third ventricle was increased in DM1 patients compared to controls (77). A second study on adult-onset DM1 and DM2 patients evaluated the echogenicities of basal ganglia and mesencephalic regions as well as ventricle diameters, however did not entirely distinguish between both disease entities. In myotonic dystrophy patients, hyperechogenicity of the substantia nigra and/or hypoechogenicity of the mesencephalic raphe were frequent findings predominantly in DM1. The width of the third ventricle was significantly larger in patients (76).

In conclusion, transcranial ultrasound B-Mode examinations are also feasible in adult-onset DM1 for the measurement of ventricular enlargement and may also detect changes in the echogenicity of specific brainstem structures.

### Myotonic dystrophy type 2

There are much more neuroimaging studies on DM1 than on DM2. The first brain imaging study on PROMM patients was performed in 1997 (7). Shortly afterwards it was discovered that PROMM and DM2 are naming the same disorder (86). Since then, a thorough Pubmed-database search revealed only further 13 neuroimaging studies and two transcranial sonography studies addressing DM2. The number of patients that were included in MRI studies were always quite low, ranging between one and 22 patients. Findings and conclusions of those studies are partly contradictory. So far, no longitudinal imaging studies on DM2 patients have been published. These are some of the reasons that explain why the natural history of brain alterations in DM2 still remains unclear.

#### Conventional morphological brain MRI in DM2

Please see Table 5 for technical details of the included studies.

**Table 5:** Conventional MRI in DM2.

**Study**	**Modality**	**Estimated parameters**	**Group size (MRI done)**	**Age (M ±SD or range) [years]**	**Age at onset [years]**	**Control group**	**Disease duration (M ±SD or range) [years]**	**Correlation parameters**
Hund et al. (7)	1.0T, 1.5T (T1, T2)	WMHL	10 (9)	27–64	26–58	-	-	-
Meola et al. (42)	1.5T (T1, PD, T2)	GCA, FCA, WMHL	20 (17)	18–73	decade 1–4	+	-	Age, NPT, neuro-muscular involvement
Kornblum et al. (24)	1.5T (T1, T2, FLAIR)	atrophy, WMHL	9 (9)	42–68	20–55	-	2–19	-
Romeo et al. (31)	1.0T T1,T2, FLAIR,DWI	WMHL	14 (12)	28–71^*^	5–67	+	16.3 ± 10.2^*^	NPT, neuro- muscular involvement
Weber et al. (33)	1.5T (T1, T2, FLAIR)	WMHL	9 (9)	53.4 ± 10.9	-	+	23.0 ± 15.0	Age, disease duration, NPT (+)
Minnerop et al. (11)	3T (T2)	WMHL	22 (22)	52.5 ± 10.1	-	+	11.9 ± 9.9	-
Schneider-Gold et al. (41)	3T (FLAIR, T1)	WMHL, VRS	16 (15)	52 ± 7^*^	24–49	+	14 ± 9^*^	Other clinical CNS symptoms

T1, T1-weighted MR sequence; T2, T2-weighted MR sequence; PD, Proton-density-weighted MR sequences; WMHL, white matter hyperintensive lesions; FCA, Focal cerebral atrophy; GCA, Global cerebral atrophy; VRS, Virchow-Robin-spaces; (+), positive or negative correlation was found;

* *data refer to whole DM2 study group, including patients that did not undergo MRI*.

Earlier studies used 1.0 or 1.5 T MRI for routine brain imaging on DM2 patients, and their main common findings were diffuse periventricular WMHL (Figure 1C) (7, 22, 24, 31, 33). From these earlier studies and those performed later using 3T-MRI (14, 41) it can be concluded that the amount of WMHL in DM2 patients is less compared to DM1 and that they are located predominantly in frontal but also in parieto-occcipital brain regions (Figure 1C). Temporal WMHL seem to be restricted to DM1 (14, 24, 31). There are contradictory assumptions regarding the lesion load, whether the lesions are confluent or non-confluent. Whereas Meola et al. and Romeo et al. did not reveal associations between WMHL and cognitive testing or neuromuscular impairment or age (22, 31), Weber et al. described that WMHL correlated with psychomotor speed (33).

### Structural brain imaging in DM2

#### Quantification of global brain volume in DM2

Please see Table 6 for technical details of the included studies.

**Table 6:** Quantification of brain volume in DM2.

**Study**	**Modality**	**Estimated parameters**	**Group size (MRI done)**	**Age (M ±SD) [years]**	**Age at onset [years]**	**Disease duration (M ±SD) [years]**	**Correlation parameters**
Kassubek et al. (52)	1.5T (T1)	BPF	9 (9)	53 ± 11	-	26 ± 16	Age (+), disease duration, motor score, educational level
Minnerop et al. (87)	1.5T (T1)	BPF VBM (WM, GM), SBM (callosal thickness)	13 (13)	53.3 ± 12.0	-	12.0 ± 8.8	-
Weber et al. (33)	1.5T (T1, T2, FLAIR)	BPF, VBM (GM)	9 (9)	53.4 ± 10.9	-	23.0 ± 15.0	Age (+), disease duration, NPT (+)
Minnerop et al. (11)	3T (T1)	VBM (GM, WM)	22	52.5 ± 10.1	-	11.9 ± 9.9	-
Franc et al. (57)	3T (T1)	DTI (GM volume)	5 (5)	38–49	29.5	-	Masticatory muscle, FA decrease (+)
Schneider-Gold et al. (41)	3T (T1)	VBM (GM, WM), volumentry (total GM/WM, cerebellum, brainstem, upper cervical cord, ventricle)	16 (15)	52 ± 7^*^	24–49	14 ± 9^*^	NPT (+), depression (+), daytime sleepiness (+), MIRS

All included studies investigated patients in comparison with controls. BPF, brain parenchymal fraction; CSF, cerebrospinal fluid; CT, cortical thickness; DTI, diffusion tensor imaging; GM, gray matter; MIRS, muscular impairment rating scale; NPT, neuropsychological tests; SBM, surface-based morphometry; VBM, voxel-based morphometry; WM, white matter; (+), positive or negative correlation was found;

* *data refer to whole DM2 study group, including patients that did not undergo MRI*.

Using routine 1.5 T brain MRI, Kornblum et al. described brain atrophy as prominent feature in DM2. Other studies also addressed this issue by applying BPF (33, 52, 87) and CNS volumetry (41). Kassubek et al. found only slightly decreased BPF in DM2 patients (52), while others (14, 33, 41), partly including larger patient groups and improved hardware, reported significant brain atrophy in DM2 patients compared to controls. However, their conclusions whether brain atrophy was driven by GM atrophy (33, 41) or WM decrease (14) remained contradictory. Kassubek et al. did not find any significant correlations of BPF in DM2 to clinical parameters (disease duration, motor score, and educational level) (52). In contrast, Weber et al. found a strong correlation of BPF to age and to visuo-constructive abilities and psychomotor speed, albeit less significant (33).

#### Quantification of regional brain volume in DM2

Please see Table 6 for technical details of the included studies.

For quantitative neuroimaging analysis a number of studies applied VBM. Minnerop et al. and Weber et al. performed VBM analyses using 1.5 T MRI in 13 and 9 DM2 patients, respectively, compared to controls (14, 87). They found cortical GM reduction, but also subcortical GM reduction in hypothalamus, thalamus, brainstem, and adjacent midline brain regions. Surprisingly, in a later study by Minnerop et al. performing VBM analyses using 3.0 T MRI in a larger group of DM2 patients no GM decrease was detected (14). Since mean age of DM2 patients was very similar across these studies, this discrepancy might be attributed to different sample sizes. However, VBM analyses revealed predominant WM alterations along corpus callosum and in every lobe, but also in the cerebellum (14, 87).

In contrast to these findings, Schneider-Gold et al. applying VBM in 16 DM2 patients in comparison to DM1 patients and controls observed the opposite relation of GM and WM effects, with a more pronounced GM loss, affecting cuneus, temporal regions, and amygdala (41). They revealed WM atrophy in the cingulate and in the subgyral WM of the medial frontal cortex and primary somatosensory cortex.

Weber et al. found hippocampal atrophy that correlated to deficits of nonverbal episodic memory in DM1 and DM2 (33). Contrary to these findings, Schneider-Gold et al. performed a range of neuropsychological tests but observed significant correlations only between flexibility of thinking and GM volume of the periaqueductal GM, midbrain, thalamus, parahippocampal gyrus, and anterior cingulate (41). Moreover, excessive daytime sleepiness was associated with GM reduction in the mediofrontal cortex and with WM reduction in the middle cerebellar peduncles and parts of pons/midbrain. Depression was associated with brainstem atrophy.

#### Quantification of white matter alterations in DM2

Please see Table 7 for technical details of the included studies.

**Table 7:** Quantification of white matter alterations in DM2.

**Study**	**Modality**	**Estimated parameters**	**Group size**	**Age (M ±SD) [years]**	**Age at onset [years]**	**Control group**	**Disease duration (M ±SD) [years]**	**Correlation parameters**
Minnerop et al. (11)	3T DTI	DTI-TBSS (FA, MD, RD, AD)	22 (22)	52.5 ± 10.1	-	+	11.9 ± 9.9	Age (+), disease duration (+), motor performance (+), depression (+), fatigue (+), NPT
Franc et al. (57)	3T DTI	DTI (FA), ROI-based	5 (5)	38–49	29.5	+	-	Masticatory muscle volume (+)

*AD, axial diffusivity; DTI, diffusion tensor imaging; FA, fractional anisotropy; MD = mean diffusivity; NPT, neuropsychological tests; RD, radial diffusivity; ROI, region of interest; TBSS, tract-based spatial statistics; (+), positive or negative correlation was found*.

DTI was applied in some DM studies in order to analyze the microstructural WM integrity. Minnerop et al. revealed WM decrease throughout the brain in DM2 patients (14). Corpus callosum was mainly affected, but also other association and projection fibers, such as internal and external capsules, and also the limbic system (fornix, cingulate bundle). However, WM decrease was less in DM2 compared to DM1 patients. Correlation analyses showed associations with age, disease duration and motor performance, also depressed mood and fatigue were associated with WM alterations in DM2. In contrast to this, no significant correlations were found between WM integrity and neuropsychological performance in DM2. In a DTI-study by Franc et al. (57) the brain was divided into compartments (supra-callosal, superior-frontal, inferior-frontal, occipital compartments). But pair-wise analyses between DM2 patients (*n* = 5) and controls did not reveal significant differences, in contrast to DM1 patients.

### Functional brain imaging in DM2

#### Positron emission tomography (PET) and single photon emission computed tomography (SPECT) in DM2

Please see Table 8 for technical details of the included studies.

**Table 8:** Functional brain imaging in DM2.

**Study**	**Modality**	**Estimated parameters**	**Group size (imaging done)**	**Age (M ±SD) [years]**	**Age at onset [years]**	**Control group**	**Disease duration (M ±SD) [years]**	**Correlation parameters**
Meola et al. (42)	H_2_O^15^-PET (resting state), SPECT	rCBF	20 (10)	18–73	decade 1–4	+	-	-
Meola et al. (88)	SPECT	CBF	19 (5)	49 ± 18	decade 2–3	+	-	-
Sansone et al. (89)	-PET (^18^F-FDG, ^11^C-β-CIT-FE, ^11^C-raclopride)	glucose metabolism, presynaptic dopamine reuptake, postsynaptic D2 receptor density	1 (1)	72	68	-	5	-
Vielhaber et al. (72)	1,5T Proton MRS	cerebral metabolism (NAA, Cho, Cr)	15 (15)	38.6 ± 7.8	>18	+	9.5 ± 6.8	-
Romeo et al. (69)	SPECT	CBF	14 (9)	54 ± ?	5–67	-	-	-
Weber et al. (33)	FDG-PET	glucose metabolism	9	53.4 ± 10.9	-	-	23.0 ± 15.0	-
Krogias et al. (76)	TCS	basal ganglia, mesencephalic regions	14 (14)	50 ± 7	9–49	+	13 ± 8	-
Rakocevic-Stojanovic et al. (90)	TCS	substantia nigra, brainstem raphe	40 (40)	51.4 ± 10.6	37.4 ± 11.1	+	14.6 ± 13.3	-
Peric et al. (68)	18F-FDG-PET	glucose metabolism	13 (13)	51.8 ± 8.4	36.5 ± 7.1	-	15.3 ± 10.5	NPT (+)

*Cho, choline; Cr, creatine; ^11^C-β-CIT-FE, ^11^C-N-(2-fluoroethyl)-2 beta-carbomethoxy-3 beta-(4-iodophenyl)nortropane; ^18^FDG, ^18^F-Fluordesoxyglucose; fMRI, functional MRI; MRS, Magnet resonance spectroscopy; PET, positron emission tomography; (r)CBF, (regional) cerebral blood flow; NAA, N-acetyl aspartate; SPECT, single photon emission computed tomography; TCS, transcranial sonography; (+), positive or negative correlation was found*.

The first functional brain imaging study on DM2 patients was performed by Meola et al. in 1999 using H_2_O^15^-PET to analyze the regional cerebral blood flow (rCBF) (22). They revealed a reduced rCBF in DM2 patients in the orbitofrontal and medial frontal cortex and discussed that this might be in accordance with deviance of behavior in DM patients.

Analyses of cerebral glucose metabolism by Weber et al. using FDG-PET on 9 DM2 patients showed significant widespread hypometabolism in frontal lobes stretching to part of the temporal lobes (33). In this study, the pattern of GM decrease (VBM) was compared to the pattern of hypometabolism (FDG-PET). Interestingly, as patterns were not conforming, it was concluded that the hypometabolism was an independent phenomenon of the disease and not a result of GM atrophy. Moreover, they did not find significant correlation of hypometabolism to neuropsychological results.

In contrast to this, a recent FDG-PET study by Peric et al. on 13 DM2 (and 16 DM1) patients observed numerous correlations with neuropsychological test results (68). They detected hypometabolism in pericentral, prefrontal, temporal regions, but also in insula, thalamus, and striatum. In DM2 they observed associations between attention deficit and prefrontal, insular and striatal hypometabolism. Executive dysfunction was associated with prefrontal and insular, right parietotemporal and frontotemporal hypometabolism.

A case report by Sansone et al. performing PET in a DM2 patient with parkinsonism revealed hypometabolism in the posterior thalamus and it was concluded, that parkinsonian features in DM2 are not a result of neurodegeneration of the nigrostriatal system but rather of hypometabolism in the posterior thalamus (89).

A neuroimaging study using single photon emission computed tomography (SPECT) by Meola et al. revealed in accordance to the PET studies, hypometabolism in frontal brain regions, but also in parietooccipital cortical regions (88).

In contrast to earlier mentioned functional brain imaging studies, Romeo et al found most significant hypoperfusion in (left) parietal lobes using perfusion SPECT on 9 DM2 patients (69).

#### Proton MR spectroscopy (^1^H-MRS) in DM2

Please see Table 8 for technical details of the study.

Vielhaber et al. used ^1^H-MRS on 15 DM2 patients, analyzing occipital and temporoparietal cortical regions and subcortical frontal WM (72). Compared to healthy controls, they revealed reduced metabolism in all tested brain regions.

#### Transcranial ultrasound in DM2

Please see Table 8 for technical details of the included studies.

In 2015 two studies using transcranial sonography on DM2 patients were published. The drawback of the study by Krogias et al. (76) was, however, that results of DM1 (*n* = 17) and DM2 (*n* = 14) patients were put together, not distinguishing entirely between both disease types. Compared to controls, transcranial sonography analyses in these patients revealed hypoechogenic signal in mesencephalon raphe and hyperechogenic signal in the substantia nigra in 29% of the patients. Looking at the separate results of both groups reveals that these sonography findings are much more frequent in DM1 than in DM2 (results for DM2: substantia nigra hyperechogenic 14.3%; mesencephalic raphe hypoechogenic 7.1%). There was a correlation between the pathological raphe signal and excessive daytime sleepiness. Moreover, they found a significant enlargement of the third ventricle in DM2 patients compared with controls.

Rakocevic-Stojanovic et al. performed transcranial sonography studies on 40 DM2 patients (90). They revealed higher frequencies of brainstem raphe hypoechogenicity and substantia nigra hyperechogenicity and increased diameter of the third ventricle (DTV). Statistical analyses revealed no correlation of substantia nigra pathology with tremor or bradykinesia in DM2 patients, and no associations of substantia nigra with depression or fatigue. In contrast to this, brainstem raphe hypoechogenicity was associated with fatigue and excessive daytime sleepiness (EDS). DTV was associated with depression and EDS. But, aberrations of brainstem raphe, substantia nigra and DTV did not correlate with sociodemographic or clinical features of DM2.

## Conclusion

### DM1

Please see Table 9 for a summary of the described regional imaging findings in DM1. Conventional MRI in DM1 patients reveals characteristic findings (e.g., atrophy, ATWML, thin corpus callosum) in particular of WM. However, only by applying more advanced and observer-independent MR methods the true extend of alteration of both WM and GM were perceived. Since then, the dependence of age at onset, the spatiotemporal evolution of brain affection, - are WM and GM changes development-related or neurodegenerative or a mixture of both—and their relevance for clinical symptoms have still been a matter of debate. Up to now, only cross-sectional studies have been published. Thus, many of the relevant questions cannot be sufficiently answered with the study results yet obtained.

**Table 9:** Summary of main regional brain changes in DM1 and DM2.

**Imaging Modality**	**DM1**	**DM2**
T1-weighted MRI	• Skull: cranial hyperostosis • Micorcephaly (congenital DM1) • Global atrophy (cortical, hippocampus, basal ganglia) • Ventricular enlargement	• Global atrophy • Ventricular enlargement
T2-weighted MRI	• WMHL (frontal and temporal lobes) • Anterior temporal white matter hyperintense lesions (ATWML) • Dilated Virchow-Robin spaces • Thinning of callosal body (congenital DM1)	• Symmetrical WMHL (periventricular, frontal, parietooccipital) • No ATWML • No dilated Virchow-Robin spaces
VBM/cortical thickness–gray matter reduction	• Cortical (all lobes) (incl. pre- and post-central gyrus, cingulate cortex, hippocampus) • Subcortical (striatum, thalamus, nucleus accumbens, cerebellum, ventral diencephalon)	• Cortical (incl. frontal, temporal, lingual gyrus, cuneus) • Subcortical (brainstem, thalamus, hypothalamus, mesencephalon, int. pallidum, amygdala)
VBM–white matter reduction	• Subcortical in all lobes • Corpus callosum • Fornix • Cingulum bundle • Subcortical (pontine, middle cerebellar peduncle, cerebellum)	• Corpus callosum • Subcortical in all lobes • Cerebellum • Cingulate
T2-relaxometry/ MTI	↑ relaxation times /↓ MT ratios: • WMHL>NAWM • Subcortical (striatum, thalamus)	-
DTI	• WMHL>NAWM • Corpus callosum • Association fibers • Limbic system tracts (fornix, cingulum bundle) • Projection fibers (internal/external capsules, corticospinal tracts) • Brainstem • Cerebellum	• WMHL>NAWM • Corpus callosum • Association fibers • Internal and external capsules • Limbic system (fornix, cingulate bundle)
PET/SPECT	*↓ FDG-Uptake*: • Cortical (frontal>temporal) • Lentiform nucleus *↓ CBF*: • Cortical (frontal>temporal) • Hypothalamus • Basal ganglia	*↓ FDG-Uptake:* • Frontal > temporal lobes • Pericentral regions • Parietal operculum • Thalamus, striatum *↓ CBF*: • Frontal, orbitofrontal, parietooccipital cortex
MR-Spectroscopy	*↓ NAA (neuronal loss)* • In WMHL und NAWM • Cortical (frontal, temporo-parietal, occipital, cingulate) *↑Cr, myo-inositol, Cho (↑glial content)* • Cortical (occcipital, temporoparietal) *↓Cr, Cho (glial loss)* • Cortical (temporoparietal, frontal)	*↓ NAA* • Cortical (occipital, temporoparietal frontal) *Cr, Cholin* • No difference compared to controls
Ultrasound	• Hypoechogenicity: brainstem raphe • Hyperechogenicity: substantia nigra • Increased width of third ventricle	• Hypoechogenicity: brainstem raphe • Hyperechogenicity: substantia nigra • Increased width of third ventricle

*CBF, cerebral blood flow; Cho, choline; Cr, creatine; DTI, diffusion tensor imaging; ^18^FDG, ^18^F-Fluordesoxyglucose; MT, magnetization transfer; MTI, magnetization transfer imaging; NAA, N-acetyl aspartate; NAWM, Normal appearing white matter; PET, positron emission tomography; SPECT, single photon emission computed tomography; VBM, voxel-based morphometry; WMHL, white matter hyperintensity lesions*.

VBM studies show a consistent widespread cortical involvement of GM affecting all lobes and in particular sensorimotor areas as well as hippocampus. Volume reductions of subcortical GM were present in striatum, thalamus and cerebellum. VBM and DTI studies investigating WM alterations found also a widespread involvement of association fibers, anterior and posterior limb of internal capsule (including the corticospinal tract), external capsule, corpus callosum, cerebellum, and brainstem.

It is well known that brain abnormalities in congenital-onset DM1 differ significantly from changes observed in patients with an adult-onset: Corpus callosum atrophy was already well known for children with a congenital disease-onset, while only by advanced MR techniques the constant and entire involvement of this structure in adult patients was recognized. Unfortunately, most of the previous studies mixed patients with different types of disease-onset. This impedes the delineation of age at onset-related patterns. Furthermore, early-onset leads to an inevitable interference with normal brain development, so it is hardly possible to distinguish pure disease-related brain changes from more secondary changes due to impaired normal brain development. Another issue is the subjective character of the clinical parameter “disease-onset” since it highly depends on the awareness of patients and their relatives to recognize disease-related symptoms at all.

In spite of these limitations a few studies compared subgroup of patients (38, 44, 57). They observed more pronounced volume reduction of GM in adult-onset DM1 patients than in patients with congenital disease-onset (38, 57), while WM alterations did not differ. Zanigni et al. even observed dissociation between cortical and subcortical GM (44): while the extent of WM alterations and subcortical GM changes remained unchanged after exclusion of patients with a congenital/childhood-onset, cortical GM changes were less pronounced than for the entire group. It could be therefore hypothesized, that the WM involvement occurs early and might be developmental, while with respect to GM involvement aging seems to play a role, pointing toward a neurodegenerative component.

Correlation analysis with CTG repeat length may be another option to obtain insights into this issue, since it is known as a relevant factor regarding disease-onset. Very long repeats (>1000) are usually associated with congenital DM1. However, measuring repeat lengths in blood as biomarker for disease severity in DM1 is highly controversial since they may not correlate with repeat lengths in brain. Somatic mosaicism of repeat expansion may lead to considerable tissue variation in repeat sizes and repeat sizes may even increase throughout life. Nevertheless, it is well accepted that the age at onset and disease severity in DM1 significantly correlate with the number of CTG repeats at least in patients with very high and very low repeat expansions.

However, although frequently performed, correlations with CTG repeat length are often negative and only observed in few studies investigating GM or WM alterations (14, 37, 40, 56). If any correlations with CTG repeat length were detected at all, it was associated with motor-related and frontal areas, further underlining the assumption that central motor areas may contribute to the motor impairment seen in DM1 patients.

Next to structural abnormalities, analyzing functional aspects of the brain is highly relevant for assessing the clinical impact and relevance to observed symptoms. PET and SPECT studies confirm the widespread cortical alteration as well as MRS studies, pointing toward neuronal and glial alterations. Correlation analyses with clinical parameters in these studies however gave heterogeneous results. The fMRI study investigating self-paced sequential finger-to thumb opposition task (32) interestingly lead to an activation of those motor-related regions that had in other DM1-studies been shown to undergo structural changes. Compensatory activations in these regions were only seen in another fMRI-study (74), analyzing the central correlates during myotonia.

The patterns in correlation analyses with other clinical parameters in structural and functional imaging studies are diverse and with the exception of few findings less reproducible. This is in particular true for correlation analysis with neuropsychological test results. Visuo-spatial functions seem to be linked to alterations of the corpus callosum and occipital areas (43, 46). Sleepiness/fatigue was linked with the volume of the ventral diencephalon (46), FA values in the brainstem (14), or MD values within association fibers (37). The heterogeneity may support the current point of view, that the clinical impairments cannot be simply linked to specific and regionally circumscribed structural or functional alterations within the brain. It seems more convincing that disturbed networks build the functional and structural substrate of clinical symptoms observed in DM1 as already seen in other neuropsychiatric diseases. Consecutively, structural and functional network analyses revealed altered functional connectivity within the default mode network in relation to personality traits/disorders in DM1, altered Theory of Mind network in relation to social cognition and detection of connectivity patterns observed in autism spectrum disorders and impaired sensorimotor networks associated with motor performance, CTG repeat length and disease duration (36, 75, 80, 82).

### DM2

Please see Table 9 for a summary of the described regional imaging findings in DM2. Routine brain MRI studies on DM2 showed primarily periventricular WMHL, and no temporal WMHL, in contrast to DM1. Further analyses revealed general brain atrophy. Most VBM studies on DM2 detected GM decrease in various cortical and brainstem regions. DTI imaging verified affected microstructural integrity predominantly of the corpus callosum, but also of numerous other association and projection fibers, including the limbic system. Overall, most of the imaging studies suggest a predominant WM disease, however data are partly contradictory. Functional brain imaging studies showed reduced perfusion and hypometabolism mainly in frontal regions, but also in temporal and parietal/parietooccipital regions. This might be associated to deficits in visuospatial and memory function and avoidant personality trait (88). Some imaging studies compared their results to clinical and neuropsychological data. The assumptions regarding clinical data (age, disease duration etc.) are contradictory. There seems to be a correlation of depression and daytime sleepiness. However, most structural MRI studies deny a correlation of brain affection and neuropsychological performance or find only few correlations with neuropsychological performance. The study by Peric et al. suggests that functional MRI might be more suitable to detect correlations of brain alterations and neuropsychological results (68). TCS studies support earlier findings of brain alterations in the brainstem and enlargement of the third ventricle.

Overall, results of these cross-sectional studies are not very consistent. Future studies need to look at a larger sample of patients. Dealing with a rare disease with diverse prevalences across different countries, multicenter studies would be needed. However, it is a big challenge to examine patients at different centers using the identical hard- and software (e.g., MRI). Moreover, no longitudinal neuroimaging studies on DM2 patients have been published so far. Thus, the natural history of brain involvement in DM2 is still unclear.

Taken together, future imaging studies in DM1 and DM2

- Should establish standard imaging procedures to facilitate the comparability across different study sites [e.g., implementation of lesion refilling tools in the VBM pipeline to avoid misclassifications of tissue classes during the segmentation step, (91)]
- Should investigate brain alterations with a multimodal approach to produce a comprehensive and versatile view of this complex and variable disorder
- Should include for DM1—if feasible—different ages at disease onset including congenital DM1 to analyze disease-related changes as a continuum
- Should have a longitudinal design to understand whether the across all studies stable and robust finding of widespread WM and GM alterations are the consequence of developmental disturbances, neurodegeneration or both
- Should clearly link functional or structural brain alterations to clinical impairment, thus facilitating the development of biomarkers for upcoming therapeutic studies

## Author contributions

All authors contributed to data collection, summary and discussion of intellectual content, manuscript writing, and editing. All authors read and approved the submitted version.

### Conflict of interest statement

The authors declare that the research was conducted in the absence of any commercial or financial relationships that could be construed as a potential conflict of interest.

## Abbreviations

AD: axial diffusivity
BPF: brain parenchymal fraction
CBF: cerebral blood flow
Cho: choline
Cr: creatine
CTG: CTG repeat length in DM1 patients
DM1: myotonic dystrophy type 1
DM2: myotonic dystrophy type 2
DMN = default mode network
DTI: diffusion tensor imaging
FA: fractional anisotropy
GM: gray matter
MD = mean diffusivity
MIRS: muscular impairment rating scale
MR: Magnetic resonance
MRS: magnetic resonance spectroscopy
NAA: N-acetyl aspartate
NAWM: Normal appearing white matter
PET: positron emission tomography
RD: radial diffusivity
ROI: region of interest
SPECT: single photon emission computed tomography
VBM: voxel-based morphometry
WM: white matter
WMHL: white matter hyperintense lesions.

## References

[1] Abe K Fujimura H Toyooka K Yorifuji S Nishikawa Y Hazama T. Involvement of the central nervous system in myotonic dystrophy. J Neurol Sci. (1994) 127:179–85. 7707077 10.1016/0022-510x(94)90071-x

[2] Pires MM Nunes B Monteiro L. Computed tomographic findings of brain and skull in myotonic dystrophy. J Neurol Neurosurg Psychiatry (1987) 50:1387–8. 10.1136/jnnp.50.10.1387-a 3681323 PMC1032474

[3] Cornacchia L Marina R Ballanini V Sozzi G. Computed tomographic findings of brain and skull in myotonic dystrophy. J Neurol Neurosurg Psychiatry (1988) 51:1463–4. 10.1136/jnnp.51.11.1463 3236032 PMC1032832

[4] Walker GL Rosser R Mastaglia FL Walton JN. Psychometric and cranial CT study in myotonic dystrophy. Clin Exp Neurol. (1984) 20:161–7. 6568937

[5] Avrahami E Katz A Bornstein N Korczyn AD. Computed tomographic findings of brain and skull in myotonic dystrophy. J Neurol Neurosurg Psychiatry (1987) 50:435–8. 10.1136/jnnp.50.4.435 3585355 PMC1031878

[6] Glantz RH Wright RB Huckman MS Garron DC Siegel IM. Central nervous system magnetic resonance imaging findings in myotonic dystrophy. Arch Neurol. (1988) 45:36–7. 10.1001/archneur.1988.00520250042017 3337674

[7] Hund E Jansen O Koch MC Ricker K Fogel W Niedermaier N. Proximal myotonic myopathy with MRI white matter abnormalities of the brain. Neurology (1997) 48:33–7. 9008490 10.1212/wnl.48.1.33

[8] Abe K Fujimura H Soga F. The fluid-attenuated inversion-recovery pulse sequence in assessment of central nervous system involvement in myotonic dystrophy. Neuroradiology (1998) 40:32–5. 10.1007/s002340050534 9493185

[9] Huber SJ Kissel JT Shuttleworth EC Chakeres DW Clapp LE Brogan MA. Magnetic resonance imaging and clinical correlates of intellectual impairment in myotonic dystrophy. Arch Neurol. (1989) 46:536–40. 10.1001/archneur.1989.00520410070026 2636847

[10] Miaux Y Chiras J Eymard B Lauriot-Prevost MC Radvanyi H Martin-Duverneuil N. Cranial MRI findings in myotonic dystrophy. Neuroradiology (1997) 39:166–70. 9106286 10.1007/s002340050385

[11] Ogata A Terae S Fujita M Tashiro K. Anterior temporal white matter lesions in myotonic dystrophy with intellectual impairment: an MRI and neuropathological study. Neuroradiology (1998) 40:411–5. 10.1007/s002340050613 9730337

[12] Di Costanzo A Di Salle F Santoro L Bonavita V Tedeschi G. Dilated Virchow-Robin spaces in myotonic dystrophy: frequency, extent and significance. Eur Neurol. (2001) 46:131–9. 10.1159/000050786 11598331

[13] Wozniak JR Mueller BA Ward EE Lim KO Day JW. White matter abnormalities and neurocognitive correlates in children and adolescents with myotonic dystrophy type 1: a diffusion tensor imaging study. Neuromuscul Disord. (2011) 21:89–96. 10.1016/j.nmd.2010.11.013 21169018 PMC3026055

[14] Minnerop M Weber B Schoene-Bake JC Roeske S Mirbach S Anspach C. The brain in myotonic dystrophy 1 and 2: evidence for a predominant white matter disease. Brain (2011) 134(Pt 12):3530–46. 10.1093/brain/awr299 22131273 PMC3235566

[15] Fiorelli M Duboc D Mazoyer BM Blin J Eymard B Fardeau M. Decreased cerebral glucose utilization in myotonic dystrophy. Neurology (1992) 42:91–4. 1734329 10.1212/wnl.42.1.91

[16] Chang L Anderson T Migneco OA Boone K Mehringer CM Villanueva-Meyer J. Cerebral abnormalities in myotonic dystrophy. Cerebral blood flow, magnetic resonance imaging, and neuropsychological tests. Arch Neurol. (1993) 50:917–23. 8363445 10.1001/archneur.1993.00540090024006

[17] Damian MS Bachmann G Herrmann D Dorndorf W. Magnetic resonance imaging of muscle and brain in myotonic dystrophy. J Neurol. (1993) 240:8–12. 10.1007/BF00838438 8423464

[18] Hashimoto T Tayama M Miyazaki M Murakawa K Kawai H Nishitani H. Neuroimaging study of myotonic dystrophy. I. Magnetic resonance imaging of the brain. Brain Dev. (1995) 17:24–7. 10.1016/0387-7604(94)00096-G 7762758

[19] Hashimoto T Tayama M Miyazaki M Murakawa K Kawai H Nishitani H. Neuroimaging study of myotonic dystrophy. II. MRI measurements of the brain. Brain Dev. (1995) 17:28–32. 10.1016/0387-7604(94)00097-H 7762759

[20] Bachmann G Damian MS Koch M Schilling G Fach B Stoppler S. The clinical and genetic correlates of MRI findings in myotonic dystrophy. Neuroradiology (1996) 38:629–35. 10.1007/s002340050322 8912317

[21] Martinello F Piazza A Pastorello E Angelini C Trevisan CP. Clinical and neuroimaging study of central nervous system in congenital myotonic dystrophy. J Neurol. (1999) 246:186–92. 10.1007/s004150050332 10323316

[22] Meola G Sansone V Perani D Colleluori A Cappa S Cotelli M. Reduced cerebral blood flow and impaired visual-spatial function in proximal myotonic myopathy. Neurology (1999) 53:1042–50. 10496264 10.1212/wnl.53.5.1042

[23] Di Costanzo A Di Salle F Santoro L Bonavita V Tedeschi G. Brain MRI features of congenital- and adult-form myotonic dystrophy type 1: case-control study. Neuromuscul Disord. (2002) 12:476–83. 10.1016/S0960-8966(01)00324-8 12031621

[24] Kornblum C Reul J Kress W Grothe C Amanatidis N Klockgether T. Cranial magnetic resonance imaging in genetically proven myotonic dystrophy type 1 and 2. J Neurol. (2004) 251:710–4. 10.1007/s00415-004-0408-1 15311347

[25] Fukuda H Horiguchi J Ono C Ohshita T Takaba J Ito K. Diffusion tensor imaging of cerebral white matter in patients with myotonic dystrophy. Acta Radiologica (2016) 46:104–9. 10.1080/02841850510015974 15841748

[26] Kuo HC Hsiao KM Chen CJ Hsieh YC Huang CC. Brain magnetic resonance image changes in a family with congenital and classic myotonic dystrophy. Brain Dev. (2005) 27:291–6. 10.1016/j.braindev.2004.09.002 15862193

[27] Giorgio A Dotti MT Battaglini M Marino S Mortilla M Stromillo ML. Cortical damage in brains of patients with adult-form of myotonic dystrophy type 1 and no or minimal MRI abnormalities. J Neurol. (2006) 253:1471–7. 10.1177/1971400915621325 16786209

[28] Di Costanzo A Santoro L de Cristofaro M Manganelli F Di Salle F Tedeschi G. Familial aggregation of white matter lesions in myotonic dystrophy type 1. Neuromuscul Disord. (2008) 18:299–305. 10.1016/j.nmd.2008.01.008 18337099

[29] Kuo HC Hsieh YC Wang HM Chuang WL Huang CC. Correlation among subcortical white matter lesions, intelligence and CTG repeat expansion in classic myotonic dystrophy type 1. Acta Neurol Scand. (2008) 117:101–7. 10.1111/j.1600-0404.2007.00911.x 18184345

[30] Kobayakawa M Tsuruya N Takeda A Suzuki A Kawamura M. Facial emotion recognition and cerebral white matter lesions in myotonic dystrophy type 1. J Neurol Sci. (2010) 290:48–51. 10.1016/j.jns.2009.11.011 20006353

[31] Romeo V Pegoraro E Ferrati C Squarzanti F Soraru G Palmieri A. Brain involvement in myotonic dystrophies: neuroimaging and neuropsychological comparative study in DM1 and DM2. J Neurol. (2010) 257:1246–55. 10.1007/s00415-010-5498-3 20221771

[32] Caramia F Mainero C Gragnani F Tinelli E Fiorelli M Ceschin V. Functional MRI changes in the central motor system in myotonic dystrophy type 1. Magn Reson Imaging (2010) 28:226–34. 10.1016/j.mri.2009.07.006 19695817

[33] Weber YG Roebling R Kassubek J Hoffmann S Rosenbohm A Wolf M. Comparative analysis of brain structure, metabolism, and cognition in myotonic dystrophy 1 and 2. Neurology (2010) 74:1108–17. 10.1212/WNL.0b013e3181d8c35f 20220122

[34] Jakkani R Jyoti S Ahmed M Thomas MM. Magnetic resonance imaging findings in adult-form myotonic dystrophy type 1. Singapore Med J. (2012) 53:e150–2. 22815033

[35] Bosemani T Jasien J Johnston MV Huisman TA Poretti A Northington FJ. Neonatal neuroimaging findings in congenital myotonic dystrophy. J Perinatol. (2014) 34:159–60. 10.1038/jp.2013.142 24476662

[36] Serra L Silvestri G Petrucci A Basile B Masciullo M Makovac E. Abnormal functional brain connectivity and personality traits in myotonic dystrophy type 1. JAMA Neurol. (2014) 71:603–11. 10.1001/jamaneurol.2014.130 24664202

[37] Wozniak JR Mueller BA Lim KO Hemmy LS Day JW. Tractography reveals diffuse white matter abnormalities in Myotonic Dystrophy Type 1. J Neurol Sci. (2014) 341:73–8. 10.1016/j.jns.2014.04.005 24768314 PMC4042407

[38] Caso F Agosta F Peric S Rakocevic-Stojanovic V Copetti M Kostic VS. Cognitive impairment in myotonic dystrophy type 1 is associated with white matter damage. PLoS ONE (2014) 9:e104697. 10.1371/journal.pone.0104697 25115999 PMC4130603

[39] Renard D Taieb G. White matter lesions in myotonic dystrophy type 1 co-locate with dilated perivascular spaces. Clin Neurol Neurosurg. (2014) 126:93–5. 10.1016/j.clineuro.2014.08.016 25218663

[40] Serra L Petrucci A Spano B Torso M Olivito G Lispi L. How genetics affects the brain to produce higher-level dysfunctions in myotonic dystrophy type 1. Funct Neurol. (2015) 30:21–31. 26214024 PMC4520669

[41] Schneider-Gold C Bellenberg B Prehn C Krogias C Schneider R Klein J. Cortical and subcortical grey and white matter atrophy in myotonic dystrophies type 1 and 2 is associated with cognitive impairment, depression and daytime sleepiness. PLoS ONE (2015) 10:e0130352. 10.1371/journal.pone.0130352 26114298 PMC4482602

[42] Conforti R de Cristofaro M Cristofano A Brogna B Sardaro A Tedeschi G. Brain MRI abnormalities in the adult form of myotonic dystrophy type 1: a longitudinal case series study. Neuroradiol J. (2016) 29:36–45. 26755488 10.1177/1971400915621325 PMC4978343

[43] Baldanzi S Cecchi P Fabbri S Pesaresi I Simoncini C Angelini C. Relationship between neuropsychological impairment and grey and white matter changes in adult-onset myotonic dystrophy type 1. Neuroimage Clin. (2016) 12:190–7. 10.1016/j.nicl.2016.06.011 27437180 PMC4939389

[44] Zanigni S Evangelisti S Giannoccaro MP Oppi F Poda R Giorgio A. Relationship of white and gray matter abnormalities to clinical and genetic features in myotonic dystrophy type 1. Neuroimage Clin. (2016) 11:678–85. 10.1016/j.nicl.2016.04.012 27330968 PMC4900512

[45] Bajrami A Azman F Yayla V Cagirici S Keskinkilic C Sozer N. MRI findings and cognitive functions in a small cohort of myotonic dystrophy type 1: retrospective analyses. Neuroradiol J. (2017) 30:23–7. 10.1177/1971400916678223 27837184 PMC5564333

[46] Cabada T Iridoy M Jerico I Lecumberri P Seijas R Gargallo A. Brain involvement in myotonic dystrophy type 1: a morphometric and diffusion tensor imaging study with neuropsychological correlation. Arch Clin Neuropsychol. (2017) 32:401–12. 10.1093/arclin/acx008 28164212

[47] Okkersen K Monckton DG Le N Tuladhar AM Raaphorst J van Engelen BGM. Brain imaging in myotonic dystrophy type 1: a systematic review. Neurology (2017) 89:960–9. 10.1212/WNL.0000000000004300 28768849

[48] Wahlund LO Barkhof F Fazekas F Bronge L Augustin M Sjogren M. A new rating scale for age-related white matter changes applicable to MRI and CT. Stroke (2001) 32:1318–22. 11387493 10.1161/01.str.32.6.1318

[49] Pantoni L Basile AM Pracucci G Asplund K Bogousslavsky J Chabriat H. Impact of age-related cerebral white matter changes on the transition to disability – the LADIS study: rationale, design and methodology. Neuroepidemiology (2005) 24:51–62. 10.1159/000081050 15459510

[50] Meola G Sansone V. Cerebral involvement in myotonic dystrophies. Muscle Nerve (2007) 36:294–306. 10.1002/mus.20800 17486579

[51] Itoh K Mitani M Kawamoto K Futamura N Funakawa I Jinnai K. Neuropathology does not correlate with regional differences in the extent of expansion of CTG repeats in the brain with myotonic dystrophy type 1. Acta Histochem Cytochem (2010) 43:149–56. 10.1267/ahc.10019 21245981 PMC3015052

[52] Kassubek J Juengling FD Hoffmann S Rosenbohm A Kurt A Jurkat-Rott K. Quantification of brain atrophy in patients with myotonic dystrophy and proximal myotonic myopathy: a controlled 3-dimensional magnetic resonance imaging study. Neurosci Lett. (2003) 348:73–6. 10.1016/s0304-3940(03)00740-7 12902021

[53] Chard DT Griffin CM Parker GJ Kapoor R Thompson AJ Miller DH. Brain atrophy in clinically early relapsing-remitting multiple sclerosis. Brain (2002) 125(Pt 2):327–37. 10.1093/brain/awf025 11844733

[54] Antonini G Mainero C Romano A Giubilei F Ceschin V Gragnani F. Cerebral atrophy in myotonic dystrophy: a voxel based morphometric study. J Neurol Neurosurg Psychiatry (2004) 75:1611–3. 10.1136/jnnp.2003.032417 15489397 PMC1738796

[55] Mathieu J Boivin H Meunier D Gaudreault M Begin P. Assessment of a disease-specific muscular impairment rating scale in myotonic dystrophy. Neurology (2001) 56:336–40. 10.1212/WNL.56.3.336 11171898

[56] Ota M Sato N Ohya Y Aoki Y Mizukami K Mori T. Relationship between diffusion tensor imaging and brain morphology in patients with myotonic dystrophy. Neurosci Lett. (2006) 407:234–9. 10.1016/j.neulet.2006.08.077 16978781

[57] Franc DT Muetzel RL Robinson PR Rodriguez CP Dalton JC Naughton CE. Cerebral and muscle MRI abnormalities in myotonic dystrophy. Neuromuscul Disord. (2012) 22:483–91. 10.1016/j.nmd.2012.01.003 22290140 PMC3350604

[58] Sugiyama A Sone D Sato N Kimura Y Ota M Maikusa N. Brain gray matter structural network in myotonic dystrophy type 1. PLoS ONE (2017) 12:e0187343. 10.1371/journal.pone.0187343 29095898 PMC5667809

[59] Hutton C Draganski B Ashburner J Weiskopf N. A comparison between voxel-based cortical thickness and voxel-based morphometry in normal aging. Neuroimage (2009) 48:371–80. 10.1016/j.neuroimage.2009.06.043 19559801 PMC2741580

[60] Naka H Imon Y Ohshita T Honjo K Kitamura T Mimori Y. Magnetization transfer measurements of cerebral white matter in patients with myotonic dystrophy. J Neurol Sci. (2002) 193:111–6. 11790391 10.1016/s0022-510x(01)00652-9

[61] Wozniak JR Mueller BA Bell CJ Muetzel RL Lim KO Day JW. Diffusion tensor imaging reveals widespread white matter abnormalities in children and adolescents with myotonic dystrophy type 1. J Neurol. (2013) 260:1122–31. 10.1007/s00415-012-6771-4 23192171 PMC3609908

[62] Di Costanzo A Di Salle F Santoro L Bonavita V Tedeschi G. T2 relaxometry of brain in myotonic dystrophy. Neuroradiology (2001) 43:198–204. 10.1007/s002340000459 11305750

[63] Winblad S Jensen C Mansson JE Samuelsson L Lindberg C. Depression in Myotonic Dystrophy type 1: clinical and neuronal correlates. Behav Brain Funct. (2010) 6:25. 10.1186/1744-9081-6-25 20482818 PMC2881877

[64] Mielke R Herholz K Fink G Ritter D Heiss WD. Positron emission tomography in myotonic dystrophy. Psychiatry Res. (1993) 50:93–9. 10.1016/0925-4927(93)90014-9 8378492

[65] Annane D Fiorelli M Mazoyer B Pappata S Eymard B Radvanyi H. Impaired cerebral glucose metabolism in myotonic dystrophy: a triplet-size dependent phenomenon. Neuromuscul Disord. (1998) 8:39–45. 9565989 10.1016/s0960-8966(97)00144-2

[66] Takeda A Kobayakawa M Suzuki A Tsuruya N Kawamura M. Lowered sensitivity to facial emotions in myotonic dystrophy type 1. J Neurol Sci. (2009) 280:35–9. 10.1016/j.jns.2009.01.014 19223261

[67] Renard D Collombier L Castelli C Pouget JP Kotzki PO Boudousq V. In myotonic dystrophy type 1 reduced FDG-uptake on FDG-PET is most severe in Brodmann area 8. BMC Neurol. (2016) 16:100. 10.1186/s12883-016-0630-3 27411408 PMC4944494

[68] Peric S Brajkovic L Belanovic B Ilic V Salak-Djokic B Basta I. Brain positron emission tomography in patients with myotonic dystrophy type 1 and type 2. J Neurol Sci. (2017) 378:187–92. 10.1016/j.jns.2017.05.013 28566162

[69] Romeo V Pegoraro E Squarzanti F Soraru G Ferrati C Ermani M. Retrospective study on PET-SPECT imaging in a large cohort of myotonic dystrophy type 1 patients. Neurol Sci. (2010) 31:757–63. 10.1007/s10072-010-0406-2 20842397

[70] Chang L Ernst T Osborn D Seltzer W Leonido-Yee M Poland RE. Proton spectroscopy in myotonic dystrophy: correlations with CTG repeats. Arch Neurol. (1998) 55:305–11. 10.1001/archneur.55.3.305 9520004

[71] Akiguchi I Nakano S Shiino A Kimura R Inubushi T Handa J. Brain proton magnetic resonance spectroscopy and brain atrophy in myotonic dystrophy. Arch Neurol. (1999) 56:325–30. 10190823 10.1001/archneur.56.3.325

[72] Vielhaber S Jakubiczka S Gaul C Schoenfeld MA Debska-Vielhaber G Zierz S. Brain 1H magnetic resonance spectroscopic differences in myotonic dystrophy type 2 and type 1. Muscle Nerve (2006) 34:145–52. 10.1002/mus.20565 16642499

[73] Takado Y Terajima K Ohkubo M Okamoto K Shimohata T Nishizawa M. Diffuse brain abnormalities in myotonic dystrophy type 1 detected by 3.0 T proton magnetic resonance spectroscopy. Eur Neurol. (2015) 73:247–56. 10.1159/000371575 25824277

[74] Toth A Lovadi E Komoly S Schwarcz A Orsi G Perlaki G. Cortical involvement during myotonia in myotonic dystrophy: an fMRI study. Acta Neurol Scand. (2015) 132:65–72. 10.1111/ane.12360 25630356

[75] Park JS Seo J Cha H Song HJ Lee SH Jang KE. Altered power spectral density in the resting-state sensorimotor network in patients with myotonic dystrophy type 1. Sci Rep. (2018) 8:987. 10.1038/s41598-018-19217-0 29343751 PMC5772436

[76] Krogias C Bellenberg B Prehn C Schneider R Meves SH Gold R. Evaluation of CNS involvement in myotonic dystrophy type 1 and type 2 by transcranial sonography. J Neurol. (2015) 262:365–74. 10.1007/s00415-014-7566-6 25385052

[77] Peric S Pavlovic A Ralic V Dobricic V Basta I Lavrnic D. Transcranial sonography in patients with myotonic dystrophy type 1. Muscle Nerve (2014) 50:278–82. 10.1002/mus.24162 24395217

[78] De Stefano N Bartolozzi ML Guidi L Stromillo ML Federico A. Magnetic resonance spectroscopy as a measure of brain damage in multiple sclerosis. J Neurol Sci. (2005) 233:203–8. 10.1016/j.jns.2005.03.018 15949506

[79] Hashimoto T Tayama M Yoshimoto T Miyazaki M Harada M Miyoshi H. Proton magnetic resonance spectroscopy of brain in congenital myotonic dystrophy. Pediatr Neurol. (1995) 12:335–40. 7546006 10.1016/0887-8994(95)00046-i

[80] Serra L Cercignani M Bruschini M Cipolotti L Mancini M Silvestri G. “I Know that You Know that I Know”: neural substrates associated with social cognition deficits in DM1 patients. PLoS ONE (2016) 11:e0156901. 10.1371/journal.pone.0156901 27258100 PMC4892543

[81] Bullmore E Sporns O. Complex brain networks: graph theoretical analysis of structural and functional systems. Nat Rev Neurosci. (2009) 10:186–98. 10.1038/nrn2575 19190637

[82] Serra L Mancini M Silvestri G Petrucci A Masciullo M Spano B. Brain connectomics' modification to clarify motor and nonmotor features of myotonic dystrophy type 1. Neural Plast. (2016) 2016:2696085. 10.1155/2016/2696085 27313901 PMC4897716

[83] Regev R de Vries LS Heckmatt JZ Dubowitz V. Cerebral ventricular dilation in congenital myotonic dystrophy. J Pediatr. (1987) 111:372–6. 10.1016/S0022-3476(87)80456-0 3305848

[84] Caglar MK Geven WB. Imaging case of the month. Cerebral ventricular dilation and diaphragmatic elevation in congenital myotonic dystrophy. Am J Perinatol. (1990) 7:198–9. 2184815 10.1055/s-2007-999480

[85] Garcia-Alix A Cabanas F Morales C Pellicer A Echevarria J Paisan L. Cerebral abnormalities in congenital myotonic dystrophy. Pediatr Neurol. (1991) 7:28–32. 2029290 10.1016/0887-8994(91)90102-q

[86] Ranum LP Rasmussen PF Benzow KA Koob MD Day JW. Genetic mapping of a second myotonic dystrophy locus. Nature Genet. (1998) 19:196–8. 10.1038/570 9620781

[87] Minnerop M Luders E Specht K Ruhlmann J Schneider-Gold C Schroder R. Grey and white matter loss along cerebral midline structures in myotonic dystrophy type 2. J Neurol. (2008) 255:1904–9. 10.1007/s00415-008-0997-1 19224318 PMC2770432

[88] Meola G Sansone V Perani D Scarone S Cappa S Dragoni C. Executive dysfunction and avoidant personality trait in myotonic dystrophy type 1 (DM-1) and in proximal myotonic myopathy (PROMM/DM-2). Neuromuscul Disord. (2003) 13:813–21. 14678804 10.1016/s0960-8966(03)00137-8

[89] Sansone V Meola G Perani D Fazio F Garibotto V Cotelli M. Glucose metabolism and dopamine PET correlates in a patient with myotonic dystrophy type 2 and parkinsonism. J Neurol Neurosurg Psychiatry (2006) 77:425–6. 10.1136/jnnp.2005.078451 16484664 PMC2077723

[90] Rakocevic-Stojanovic V Peric S Savic-Pavicevic D Pesovic J Mesaros S Lavrnic D. Brain sonography insight into the midbrain in myotonic dystrophy type 2. Muscle Nerve (2016) 53:700–4. 10.1002/mus.24927 26425828

[91] Ceccarelli A Jackson JS Tauhid S Arora A Gorky J Dell'Oglio E. The impact of lesion in-painting and registration methods on voxel-based morphometry in detecting regional cerebral gray matter atrophy in multiple sclerosis. AJNR Am J Neuroradiol. (2012) 33:1579–85. 10.3174/ajnr.A3083 22460341 PMC3425668

